# Supplementary material for: Risk of atrial fibrillation and association with other diseases: protocol of the derivation and international external validation of a prediction model using nationwide population-based electronic health records
Source: BMJ Open. 2023 Dec 9;13(12):e075196. doi: 10.1136/bmjopen-2023-075196 (PMC10729260; doi:10.1136/bmjopen-2023-075196)
Supplement: Supplementary data [file bmjopen-2023-075196supp001.pdf]

Supplementary Appendix

Risk of atrial fibrillation and association with other diseases: protocol of the derivation and international external validation of a prediction model using nationwide population-based electronic health records

Ramesh Nadarajah, Jianhua Wu, Ronen Arbel, Moti Haim, Doron Zahger, Talish Razi Benita, Lior Rokach, Campbell Cowan, Chris P Gale

Supplementary Table 1. Baseline demographic and comorbidity variables used in algorithms tested for predicting incident AF in community-based electronic health records 2

Supplementary Table 2. Definition of disease categories for causes of deaths 4

Supplementary Figure 1. Design process leading to selection of non-AF outcomes to assess for association with predicted AF risk 5

Supplementary Table 1. Baseline demographic and comorbidity variables used in algorithms tested for predicting incident AF in community-based electronic health records

Algorithm	Demographics	Comorbidities
$CHADS_2$	Age	Hypertension, CHF, diabetes mellitus, CVA
CHA_2DS_2-VASc	Age, sex	Hypertension, CHF, stroke/TIA/thromboembolism, vascular disease
CHARGE-AF	Age, race, smoking status	Anti-hypertensive medication, MI, CHF, DM
C_2HEST	Age	Hypertension, ischaemic heart disease, CHF, COPD, thyroid disease
HATCH	Age	Hypertension, CHF, stroke/TIA, COPD
InGef	Age, sex	Anti-hypertension medication, heart failure medication, chronic kidney disease, disorderd of lipoprotein metabolism and other lipidaemias, pulmonary heart diseases cardiac arrhythmias, other cerebrovascular disease, diverticular disease of intestine, dorsalgia, breathing abnormalities
MHS	Age, sex	Anti-hypertensive medication, MI, CHF, peripheral vascular disease, inflammatory disease in a female, COPD
NHIRD	Age (years), age group, sex	Hypertension, CHF, COPD, rheumatological disease, dyslipidaemia, DM, CVA or TIA, sleep disorder, cancer, hyperthyroidism, vascular disease, gout, CKD or ESRD, anaemia
NHIS-NSC*	Age, sex, smoking (pack-year), alcohol	Hypertension, CHF, MI, vascular disease, stroke/TIA, COPD
Pfizer-AI	Age, sex, race, smoking status	Hypertension, anti-hypertensive medication, CHF, congenital heart disease, MI, LVH, type 1 DM, type 2 DM
Taiwan AF	Age, sex, alcohol excess	Hypertension, CHF, IHD, ESRD

AF, Atrial Fibrillation; $CHADS_2$, Congestive heart failure, Hypertension, Age >75, Diabetes mellitus, prior Stroke or transient ischemic attack [2 points]; CHA_2DS_2-VASc, Congestive heart failure, Hypertension, Age >75 [2 points], Stroke/transient ischemic attack/thromboembolism [2 points]; CHARGE-AF, Cohorts for Heart and Aging Research in Genomic Epidemiology; C_2HEST, Coronary artery disease / Chronic obstructive pulmonary disease [1 point each], Hypertension, Elderly (Age ≥75, 2 points), Systolic heart failure, Thyroid disease (hyperthyroidism); CHF, chronic heart failure; CKD, chronic kidney disease; COPD, chronic obstructive pulmonary disease; CPRD, Clinical Practice Research Datalink; CVA, cerebrovascular accident; DM, diabetes mellitus; ESRD, end-stage renal disease; HATCH, Hypertension, Age, stroke or Transient ischemic attack, Chronic obstructive pulmonary disease, Heart failure; IHD, ischaemic heart disease; LVH, left ventricular hypertrophy; MHS, Maccabi Healthcare Services; MI, myocardial infarction; NHIRD, National Health Insurance Research Database; NHIS-HEALS, National Health Insurance Service - Health screening Cohort; NHIS-NSC, National Health Insurance Service-based National Sample Cohort; TIA, transient ischaemic attack.

* In Kim 2020 prediction model development using machine learning was completed both with and without the predictor $PM_{2.5}$ - which is fine particular matter air pollution. In this analysis we have only included the model without $PM_{2.5}$ as it is judged not to be a predictor that would be routinely available in primary care or population EHR.

Supplementary Table 2. Definition of disease categories for causes of deaths

Causes of death	Code
**Cardiovascular disorders**	ICD chapter 'Diseases of the circulatory system' (code range: I00–I99), excluding codes relating to infections or cerebrovascular disease.
**Cerebrovascular disorders**	ICD chapter 'Diseases of the circulatory system' (I60-I69)
**Neoplasms**	ICD chapter 'Neoplasms' (C00–D48).
**Infections**	Infectious and parasitic diseases, respiratory infections, urinary tract infections, and cellulitis, as defined by individual codes as Conrad et al.
**Chronic respiratory diseases**	Individual codes Conrad et al.
**Digestive diseases**	ICD chapter 'Diseases of the digestive system' (K00–K93), excepting selected codes categorized as infections.
**Mental and neurological disorders**	ICD chapter 'Mental and behavioral disorders' (F00–F99) and ICD chapter 'Diseases of the nervous system' (G00–G99)
**Injuries**	ICD chapters 'Injury, poisoning and certain other consequences of external causes' (S00–T98) and 'External causes of morbidity and mortality' (V01–Y98)
**Kidney diseases**	ICD sub-chapters 'Renal failure' (N17-N19), 'Glomerular diseases' (N00-N08), 'Renal tubulo-interstitial diseases' (N10-N16), 'Other disorders of kidney and ureter' (N25-N29)

To categorise cause of death as infections or chronic respiratory diseases we used the same codelists as Conrad N, Judge A, Canoy D, et al. Temporal trends and patterns in mortality after incident heart failure: a longitudinal analysis of 86 000 individuals. *JAMA cardiology* 2019;4(11):1102-11

Supplementary Figure 1. Design process leading to selection of non-AF outcomes to assess for association with predicted AF risk

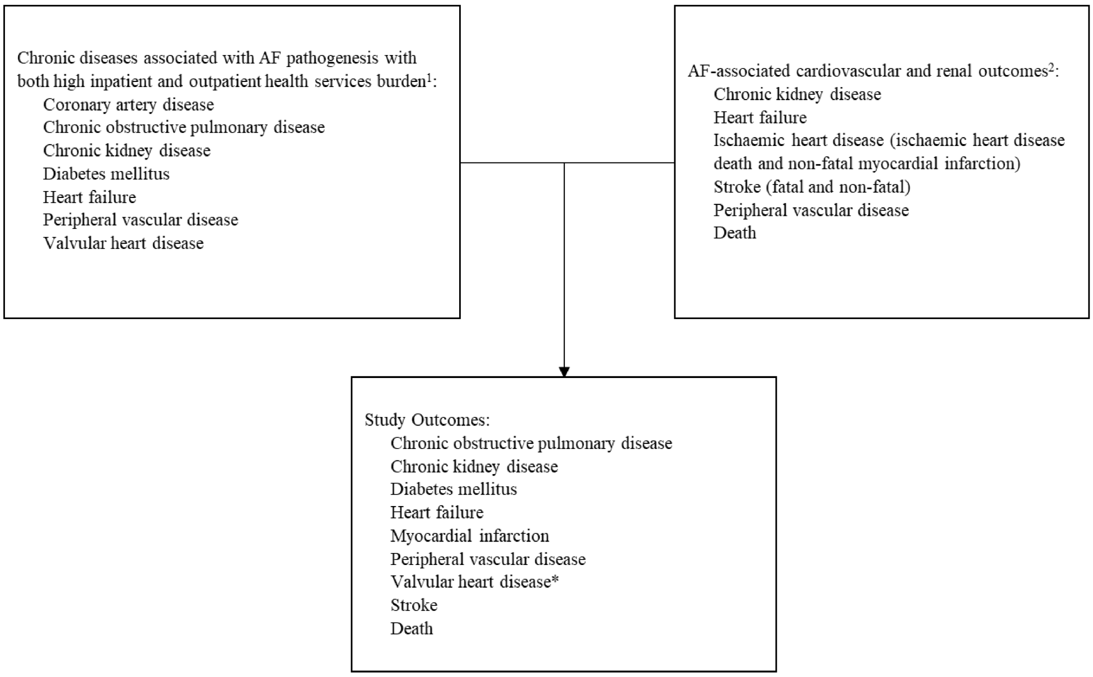

[1] Hindricks G, Potpara T, Dagres N, et al. 2020 ESC Guidelines for the diagnosis and management of atrial fibrillation developed in collaboration with the European Association for Cardio-Thoracic Surgery (EACTS) The Task Force for the diagnosis and management of atrial fibrillation of the European Society of Cardiology (ESC) Developed with the special contribution of the European Heart Rhythm Association (EHRA) of the ESC. 2021;42(5):373-498.

[2] Odutayo A, Wong CX, Hsiao AJ, et al. Atrial fibrillation and risks of cardiovascular disease, renal disease, and death: systematic review and meta-analysis. *BMJ* 2016;354

* Aortic stenosis was further specified in addition to valvular heart disease given the increasing availability and randomised controlled trial evidence for earlier treatment, and increasing therapeutic options across operative risk profiles (Vahanian A, Beyersdorf F, Praz F, et al. 2021 ESC/EACTS Guidelines for the management of valvular heart disease: developed by the Task Force for the management of valvular heart disease of the European Society of Cardiology (ESC) and the European Association for Cardio-Thoracic Surgery (EACTS). *Eur Heart J* 2022;43(7):561-632.)
