# Supplementary material for: Risk of atrial fibrillation and association with other diseases: protocol of the derivation and international external validation of a prediction model using nationwide population-based electronic health records
Source: BMJ Open. 2023 Dec 9;13(12):e075196. doi: 10.1136/bmjopen-2023-075196 (PMC10729260; doi:10.1136/bmjopen-2023-075196)
Supplement: Author's manuscript [file bmjopen-2023-075196.draft_revisions.pdf]

BMJ Open is committed to open peer review. As part of this commitment we make the peer review history of every article we publish publicly available.

When an article is published we post the peer reviewers' comments and the authors' responses online. We also post the versions of the paper that were used during peer review. These are the versions that the peer review comments apply to.

The versions of the paper that follow are the versions that were submitted during the peer review process. They are not the versions of record or the final published versions. They should not be cited or distributed as the published version of this manuscript.

BMJ Open is an open access journal and the full, final, typeset and author-corrected version of record of the manuscript is available on our site with no access controls, subscription charges or pay-per-view fees (http://bmjopen.bmj.com).

If you have any questions on BMJ Open's open peer review process please email info.bmjopen@bmj.com

BMJ Open

Risk of atrial fibrillation and association with other diseases: protocol of the derivation and international external validation of a prediction model using nationwide population-based electronic health records

Journal:	*BMJ Open*
Manuscript ID	bmjopen-2023-075196
Article Type:	Protocol
Date Submitted by the Author:	28-Apr-2023
Complete List of Authors:	Nadarajah, Ramesh; University of Leeds, Leeds Institute of Data Analytics; Leeds Teaching Hospitals NHS Trust, Department of Cardiology<br>Wu, Jianhua; University of Leeds, Leeds Institute for Data Analytics; Queen Mary University of London, Wolfson Institute of Population Health<br>Arbel, Ronen; Ben-Gurion University of the Negev, Health Systems Management; Sapir College,<br>Haim, Moti; Soroka University Medical Center, Department of Cardiology; Ben-Gurion University of the Negev<br>Zahger , Doron ; Soroka University Medical Center<br>Benita, Talish Razi; Clalit Health Services; Ben-Gurion University of the Negev<br>Rokach, Lior; Ben-Gurion University of the Negev, Department of Information Systems and Software Engineering<br>Cowan, Campbell; Leeds Teaching Hospitals NHS Trust, Department of Cardiology<br>Gale, Chris; University of Leeds
Keywords:	Electronic Health Records, Pacing & electrophysiology < CARDIOLOGY, PREVENTIVE MEDICINE, Primary Care < Primary Health Care

SCHOLARONE™
Manuscripts

Title:

Risk of atrial fibrillation and association with other diseases: protocol of the derivation and international external validation of a prediction model using nationwide population-based electronic health records

Publication type: Study Protocol

Target journal: BMJ Open

Authors:

Ramesh Nadarajah[1,2,3], Jianhua Wu[4,5], Ronen Arbel[6,7], Moti Haim[8,9], Doron Zahger[10,11], Talish Razi Benita[6,9], Lior Rokach[10], Campbell Cowan[3], Chris P Gale[1,2,3]

Affiliations:

[1] Leeds Institute for Cardiovascular and Metabolic Medicine, University of Leeds, UK

[2] Leeds Institute of Data Analytics, University of Leeds, UK

[3] Department of Cardiology, Leeds Teaching Hospitals NHS Trust, Leeds, UK

[4] Wolfson Institute of Population Health, Queen Mary, University of London, UK

[5] School of Dentistry, University of Leeds, Leeds, UK

[6] Community Medical Services Division, Clalit Health Services, Tel Aviv, Israel

[7] Maximizing Health Outcomes Research Lab, Sapir College, Sderot, Israel

[8] Department of Cardiology, Soroka University Medical Center, Beer Sheva, Israel

[9] Faculty of Health Sciences, Ben Gurion University of the Negev, Beer Sheva, Israel

[10] Department of Information Systems and Software Engineering, Ben Gurion University of the Negev, Beer-Sheva, Israel

For peer review only

Correspondence:

Ramesh Nadarajah

British Heart Foundation Clinical Research Fellow

Leeds Institute for Cardiovascular and Metabolic Medicine

University of Leeds

6 Clarendon Way

Leeds, UK

LS2 9DA

Tel +44 (0) 113 343 3241

Email r.nadarajah@leeds.ac.uk

Twitter @Dr_R_Nadarajah

Word Count

3911

Keywords

Atrial Fibrillation

Prediction

Screening

Community

Electronic health records

Cardiometabolic

Outcomes

For peer review only

Risk

Population

For peer review only

Abstract

Introduction

Atrial fibrillation (AF) is a major public health issue and there is rationale for the early diagnosis of AF, before the first complication occurs. Previous AF screening research is limited by low yields of new cases and strokes prevented in the screened populations. For AF screening to be clinically and cost-effective, the efficieny of identification of newly diagnosed AF needs to be improved and the intervention offered may have to extend beyond oral anticoagulation for stroke prophylaxis. Previous prediction models for incident AF have been limited by their data sources and methodologies.

Methods and analysis

We will investigate the application of Random Forest and multivariable logistic regression to predict incident AF within a 6 months prediction horizon, that is a time-window consistent with conducting investigation for AF. The Clinical Practice Research Datalink (CPRD)-GOLD dataset will be used for derivation, and the Clalit Health Services dataset will be used for international external geographical validation. Analyses will include metrics of prediction performance and clinical utility. We will create Kaplan-Meier plots for individuals identified as higher and lower predicted risk of AF and derive the cumulative incidence rate for non-AF cardio-renal-metabolic diseases and death over the longer term to establish how predicted AF risk is associated with a range of new non-AF disease states.

Ethics and dissemination

Permission for CPRD-GOLD was obtained from CPRD (ref no: 19_076). The CPRD ethical approval committee approved the study. CHS Helsinki committee approval 21-0169 and data

For peer review only

utilization committee approval 901. The results will be submitted as a research paper for publication to a peer-reviewed journal and presented at peer-reviewed conferences.

Trial registration details

A systematic review to guide the overall project was registered on PROSPERO (registration number CRD42021245093). The study was registered on Clinical Trials.gov (NCT05837364).

For peer review only

Strengths and limitations of the study

- Large and nationwide datasets representative of the community-dwelling populations in two countries.
- Predicting risk of incident AF in the short-term may be more useful to screening than longer prediction horizons
- Quantification of the strength of association between predicted AF risk and other diseases may uncover other opportunities that could be actioned during AF screening beyond stroke prophylaxis.
- The derivation and validation work will be undertaken in datasets in the UK and Israel; therefore, further validation work may be pursued with newly-collected data and for other contexts.
- The derivation data will not include unstructured natural language free text; future research could explore if incorporating free text improves predictive accuracy.
- A calculator created from a parsimonious model may enhance the usability of the model in the real world and in contexts where electronic health records are unavailable or incomplete.

For peer review only

INTRODUCTION

Atrial fibrillation (AF) is the most common sustained cardiac arrhythmia. Over the last 20 years the number of new cases of AF diagnosed each year has risen by 72%, and now surpasses the four most common causes of cancer combined.[1] Moreover, it is estimated that up to 35% of disease burden remains undiagnosed,[2] and 15% of strokes occur in the context of undiagnosed AF.[3]

Oral anticoagulants can reduce the risk of stroke by up to two thirds in those with AF at higher risk of stroke,[4] and international guidelines recommend their use in patients with AF at elevated thromboembolic risk.[5] Early detection of AF may permit the initiation of oral anticoagulation to reduce embolic stroke risk,[6] and early antiarrhythmic therapy to reduce the risk of death and stroke.[7] Accordingly early AF detection is a key cardiovascular priority in the UK NHS Long Term Plan,[8] and the European Society of Cardiology recommends opportunistic screening by pulse palpation or electrocardiogram (ECG) rhythm strip in persons aged ≥65 years and systematic ECG screening in those aged ≥75 years.[9]

Furthermore, AF frequently develops due to, and in parallel with, other cardiovascular, renal and metabolic conditions,[10] and individuals with AF are at an increased risk of major cardiovascular events in excess of stroke including ischemic heart disease, heart failure, chronic kidney disease, peripheral vascular disease and death.[11] Thus, AF screening, with or without AF diagnosis, may be a key opportunity for holistic management of cardiometabolic risk factors and unhealthy lifestyle behaviours to reduce an individual's risk of later adverse events beyond that of stroke prophylaxis alone.

For peer review only

Several studies have shown that serial or continuous non-invasive electrocardiogram (ECG) monitoring in older people with stroke risk factors / elevated N-terminal pro B-type natriuretic peptide (NT-proBNP), leads to a higher detection rate of previously undiagnosed AF compared with routine standard of care, though yields remain relatively low (3.0%-4.4%).[12-15] The STROKESTOP randomised controlled trial, where AF screening was offered to individuals aged 75 and 76 years without exclsions, achieved only a 3% yield of new AF cases with a modest benefit in a composite outcome of ischaemic or haemorrhagic stroke, systemic embolism, bleeding leading to hospitalisation and all-cause death; and not for each of ischaemic stroke, haemorrhagic stroke, or hospitalisation for major bleeding.[16] Accordingly, for AF screening to be effective the yield of newly diagnosed AF amongst participants needs to be improved and the intervention offered may have to extend beyond only oral anticoagulation for stroke prophylaxis (Figure 1).

A large proportion of the population are registered in primary care with a routinely-collected electronic health record (EHR).[17 18] A prediction model that utilises data available in the community to calculate AF risk could discriminate patients into risk categories, with screening offered only to higher risk individuals,[19] enabling scalable and efficient targeted AF screening. To date, several multivariable prediction models have been created or tested for prediction of incident AF in community-based electronic health records, but are of limited clinical utility for AF screening on account of moderate discriminative performance, long prediction horizons and limited scalability due to missing data.[20] None have yet reached widespread clinical practice. Moreover, reports of prediction models have yet to quantify the association between AF risk and new disease states outside that of AF and stroke.

For peer review only

Research aim

The aims of this study are to:

1) Develop a model for predicting short-term AF risk from data routinely available in community-based EHRs.
2) Quantify the association of predicted AF risk with a range of non-AF diseases.
3) Externally validate the prediction model in an international context to assess transportability.
4) Produce a calculator derived from a parsimonious prediction model.

For peer review only

METHODS AND ANALYSIS

Data sources and permissions

The derivation dataset will be the Clinical Practice Research Datalink-GOLD (CPRD-GOLD) dataset. This is an ongoing primary care database, established in 1987, that comprises anonymised medical records and prescribing data contributed by general practices using Vision® software. It contains data for approximately 17.5 million patients, with 30% of contributing practices in England, and represents the United Kingdom (UK) population in terms of age, sex and ethnicity.[17] In order to contribute to the database, general practices and other health centres must meet prespecified standards for research-quality data ('up-to-standard').[17 21]

Recorded information includes patients' demography, clinical symptoms, signs, investigations, diagnoses, prescriptions, referrals, behavioural factors and test results entered by clinicians and other practice staff. All clinical information is coded using Read Codes.[22] Extracted patients will have patient-level data linked to Hospital Episode Statistics (HES) Admitted Patient Care (APC) and Office for National statistics (ONS) Death Registration. The CPRD dataset has been used to develop or validate a range of risk prediction models, including in cardiovascular disease.[23]

The extracted dataset, including linked data, comprises all patients for the period between 2nd January 1998 and 30th November 2018 from the snapshot of CPRD-GOLD in October 2019. Over this study period, the CPRD-GOLD dataset comprises approximately 2 million patients eligible for data linkage at an up-to-standard practice, with over 200,000 patients having a record of AF during follow-up.

For peer review only

To ascertain whether the prediction model is transportable to geographies outside of the UK, we will externally validate its performance in the Clalit Health Services database in Israel. As a result of the National Health Insurance Law, Israeli citizens are required to enroll in 1 of 4 payer-provider health funds and receive free basic health care. Clalit Health Services (CHS) provides health insurance coverage to 4.8 million insured members, and about two thirds of the population aged >65 years. CHS is recooginzed globally as the primary source of evaluation of Covid-19 vaccinations and therapies.[24-27] All clinical information is coded in International Classifications of Diseases, Ninth Revision (ICD-9). Receipt of vital status from the Ministry of the Interior ensures 100% follow-up of mortality. We will include participants insured by Clalit with continuous membership for at least 1 year before 01/01/2019: 2,159,663 patients with 4,330 of them having a new icident of AF (Atrial fibrillation and/or atrial flutter) in the first half of 2019.

Patient and Public Involvement

The Arrhythmia Alliance and AF association provided input on the FIND-AF scientific advisory board. The FIND-AF patient and public involvement group have given input to reporting and dissemination plans of the research.

Inclusion and exclusion criteria

The study population for derivration and internal validation will comprise all available patients in CPRD-GOLD eligible for data linkage and with at least 1-year follow-up in the period between 2nd January 1998 and 30th November 2018. For the external validation the study population will comprise participants insured by CHS, including those with continuous membership for at least 1 year, before 01/01/2019 . Patients will be excluded if they were ≤30 years of age, or diagnosed with AF or atrial flutter (AFl) at the point of study entry, registered

For peer review only

for less than 1 year or, in CPRD, ineligible for data linkage. Patients younger than 30 years of age are not included in the cohort for AF prediction because the incidence of AF over even a 10-year horizon is very low in this group.[1]

Prediction model outcome ascertainment

The outcome of interest is first diagnosed AF or AFl after baseline. We have included AFl as an outcome since it has similar clinical relevance, including thromboembolic risk and anticoagulation guidelines, as AF.[5] These will be identified using Read codes in CPRD dataset. For HES APC events and underlying cause of death variable in the ONS Death Registration data file, ICD-10 codes will be used. For CHS events will be identified using ICD-9 codes.

Sample size

To develop a prognostic prediction model, the required sample size may be determined by three criteria suggested by *Riley et al*.[28] For example, suppose a maximum of 200 parameters will be included in the prediction model and the Cox-Snell generalised R^2 is assumed to be 0.01. A total of 377,996 patients will be required to meet Riley's criterion (i) with global shrinkage factor of 0.95; this sample size also ensures a small absolute difference ($\Delta < 0.05$) in the apparent and adjusted Nagelkerke R^2 (Riley's criterion (ii)) and ensures precise estimate of overall risk with a margin of error < 0.001 (Riley's criterion (iii)). According to the Quality and Outcomes Framework (QOF), the prevalence of AF in England is 1.7%.[29 30] Given an AF prevalence of 1.7%, only 6,425 patients will be expected to develop AF from 377,996 patients. Within the Clalit Health Services database there are 2,159,663 patients. Therefore, the number of patients in the CPRD and Clalit health services datasets with AF will provide sufficient statistical power to develop and validate a prediction model with the predefined precision and accuracy.

For peer review only - http://bmjopen.bmj.com/site/about/guidelines.xhtml

Predictor Variables

A systematic review has been conducted to establish predictor variables included in varying combinations by preceding prediction models developed to detect incident AF in community-based EHRs (Supplementary Table 1),[31] and supplemented with a literature search for variables associated with incident AF.

Candidate variables include

1. Sociodemographic variables including age, sex and ethnicity (SocioEconomic Score and population sector will serve as surrogate for ethnicity in CHS)
2. All disease conditions during follow-up, including hospitalised diseases and procedures, such as other cardiovascular diseases, diabetes mellitus, chronic lung disease, renal disease, inflammatory disease, cancer, hypothyroidism and surgical procedures.
3. Lifestyle factors including smoking status and alcohol consumption that are coded in structured Read codes.

Predictive factors will be identified using the appropriate codes, with Read codes for diagnoses and lifestyle factors. Code lists for predictors will be used from publications if available, otherwise the CPRD code browser will be used and codes checked by at least two clinicians. The code lists for predictors in CPRD-GOLD will be adapted from CALIBER and HDR UK repositories or publications. If none are available from these sources then new code lists developed using the OpenCodelists and checked by at least two clinicians. Diagnostic code lists will comprise the primary care coding system (Read codes), to ensure that only information readily available within a primary care EHR could be incorporated within the

For peer review only

prediction model. Within CHS, the code lists for predictors will be developed using similar methods based on the medical records and coding of CHS, which also includes a validated chronic diseases registry.

Candidate variable data types are deliberately limited to ensure widespread applicability of the model given the reality of 'missing' data in routinely-collected electronic health records.[18] Observations and laboratory results are not included. Ethnicity information is routinely collected in the UK NHS and so has increasingly high completeness,[32] and we will include an 'ethnicity unrecorded' category where it is unavailable because missingness is considered informative.[33] Ethicity in a UK context does not directly translate to an Israeli context so sociodemographic surrgoates will be used: i) .population sectors- General Jewish, ultra-orthodox Jewish and Arab ii). Socioeconomic score on a scale of 1-10. For diagnoses, if medical codes are absent in a patient record we will assume that the patient does not have that diagnosis, or that the diagnosis was not considered sufficiently important to have been recorded by the GP in case of symptoms.[35] Concordantly, the analytical cohorts are not expected to have missing data for any of the predictor variables.

<u>Data analysis plan</u>

Data pre-processing

The CPRD-GOLD and Clalit Health Services data will be cleaned and preprocessed for model development, internal validation and external validation. Specifically, for patient features with binary values, 0 and 1 will be mapped to the binary values. Variables with multiple categories (ethnicity) will be split into their component categories, and each given a binary value to indicate the presence or not of the variable for each patient. Continuous variables (age) will be kept as continuous.

For peer review only

Descriptive analysis

Continuous variables will be reported as mean ± standard deviation (SD) and categorical variables as frequencies with corresponding percentages.

Prediction model development

We will compare a machine learning and logistic regression approach to prediction model development for incident AF in CPRD-GOLD. Logistic regression model offers a more manageable approach for implementation, interpretation and training compared to machine learning algorithms, but machine learning methods can better handle non-linearities and interactions among variables and may lead to better discriminative performance.[20]

We will investigate the use of a random forest classifier for AF prediction in the CPRD-GOLD dataset. In our systematic review of AF prediction in EHRs it had the most evidence for use and showed robust performance in different datasets and geographies.[20] Random Forest (RF) is an ensemble technique that combines a large number of decision trees using a bagging approach to improve the overall performance (Figure 2).[34] In brief, the bagging approach grows multiple classification trees in parallel where each tree gives a classification which are called votes. These votes are then aggregated to provide a more accurate and stable prediction. Furthermore the degree of variation of each feature in a RF classifier for the prediction task can be calculated using the mean decrease in the Gini coefficient, a measure of how each variable contributes to the homogeneity of nodes and leaves in the resulting RF. Showing the importance of variables used in prediction (explainability) is considered important for clinical uptake of prediction models,[35] and a limitation of using deep learning techniques.

For peer review only

Preprocessed patient-level data in CPRD-GOLD will be randomly split into an 80:20 ratio to create derivation and internal validation (or training and testing) samples. The split ratio is not a significant factor, given the volume of the sample size. The model parameters and dropout rate, will be chosen through a grid search and 10-fold cross-validation will be used (i.e. 10% of the training data will be randomly selected as the cross-validation set). The multivariable logistic regression model will be developed with backward model selection with Akaike information criterion.[36] The prediction window will be set at 6 months, as this is considered in keeping with the logistical time frames for organising AF investigation at scale.[37]

Internal validation

We will evaluate the model performance using a validation cohort with internal bootstrap validation with 200 samples. The AUROC will be used to evaluate predictive ability (concordance index) with 95% confidence intervals calculated using the DeLong method.[38] Youden's index will be established for the outcome measure as a method of empirically identifying the optimal dichotomous cut-off to assess sensitivity, specificity, positive predictive value and negative predictive value. We will calculate the Brier score, a measure of both discrimination and calibration, by taking the mean squared difference between predicted probabilities and the observed outcome. To assess the clinical impact of utilising FIND-AF as opposed to other risk prediction scores, we will calculate the net reclassification index at the risk threshold that equates to the average 6 months incidence rate in the cohort and conduct a decision curve analysis, which assesses across threshold probabilities whether the predictive model would do more benefit than harm. Calibration will be assessed graphically by plotting predicted AF risk against observed AF incidence and quantified using a calibration slope.

For peer review only

The same methods will be employed in subgroups by age (<65 years, ≥65 years, <75 years, ≥75 years), sex (women, men) and ethnicity (White, Black, Asian, others and unspecified) to assess the model's predictive performance across clinically relevant groups.

Performance of the prediction model will be compared with the CHA_2DS_2-VASc and C_2HEST scores. The CHA_2DS_2-VASc score was originally developed to predict stroke risk in individuals with AF, and the C_2HEST score for Asian people without structural heart disease.[20] These algorithms are robust to missing data in routinely-collected primary care EHRs and have been tested for AF risk prediction in European cohorts.[20] Other algorithms that can only be applied to a minority of European primary care EHRs (Pfizer-AI, CHARGE-AF) will not be considered as they cannot be implemented at scale to inform AF screening.[18 37]

Quantification of the association between short-term predicted AF risk and long-term AF and other diseases

We will include all patients randomly assigned to the testing dataset in CPRD-GOLD by the Mersenne twister pseudorandom number generator, categorized as lower or higher predicted AF risk by the developed prediction model. For long-term AF risk we will plot Kaplan-Meier plots for individuals identified as higher and lower predicted risk of AF to assess the event rate for AF censored at 10 years, and calculate the hazard ratio for AF between higher and lower predicted risk of AF using the Cox proportional hazard model with adjustment for the competing risk of death. This will inform us of whether short-term AF risk is also associated with long-term AF risk, and whether an individual who undergoes risk-guided AF screening should be considered for repeated AF screening at a later time point (e.g. 1 or 5-years).

For peer review only

For non-AF disease states we will consider the initial presentation of a cardiovascular, renal, or metabolic disease or death. This is because AF is not a disease in isolation and is known to be associated with high risk of adverse clinical outcomes. To best characterise highly prevalent and morbid diseases, associated with the development or consequence of AF and that may be appropriate for prevention or targeted diagnostic pathways subsequent to AF screening (Supplementary Figure 1),[10] we will individually examine the following nine conditions: heart failure, valvular heart disease (and specifically aortic stenosis), myocardial infarction, stroke (ischaemic and haemorrhagic) or transient ischaemic attack, peripheral vascular disease, chronic kidney disease, diabetes mellitus, as well as chronic obstructive pulmonary disease (COPD). These disease states have been further selected for investigation because interventions could be implemtented and / or tested to reduced their clinical progression. We will also quantify the occurrence of death by any cause recorded in primary care or by death certification from the UK Death Register of the Office for National Statistics, which will be mapped on to 9 disease categories (Supplementary Table 2). For each condition, a list of diagnostic codes from the CALIBER code repository, including from International Classification of Diseases 10th revision (used in secondary care) and Read coding schemes (used in primary care) will be defined to comprehensively to identify diagnoses from EHRs. Incident diagnoses will be defined as the first record of that condition in primary or secondary care records from any diagnostic position. For definition of new cases, we will exclude individuals for the analysis of each condition who had a diagnosis of that condition before the patient's entry to the study. If no indication of a specific disease is recorded, then the patient will be assumed to be free from the disease. CPRD is a positive recording dataset, which reduces the likelihood of the non-recording of a clinically identified disease state.

For peer review only

We will create Kaplan-Meier plots for individuals identified as higher and lower predicted risk of AF and derive the cumulative incidence rate for each outcome at 1, 5 and 10 years considering the competing risk of death, as well as death at 5 and 10 years. For each specified outcome, we will calculate the hazard ratio (HR) between higher and lower predicted risk of AF using the Fine and Gray's model with adjustment for the competing risk of death. We will also report adjusted HR where the model is adjusted for age, sex, ethnicity and the presence of any of the other outcomes at baseline. As some of the outcomes have incidence rates that are strongly associated with age (e.g. aortic stenosis) or differ by sex (e.g. heart failure),[38 39] we will conduct sub-group analyses of incidence rates for higher and lower risk individuals for each outcome by age group (30 to 64 years and ≥65 years) and sex. As some of the non-AF outcomes are more likely to occur in the setting of prevalent AF (e.g. stroke or heart failure),[10] we will also conduct a sensitivity analysis whereby people with incident AF during follow-up are excluded.

External validation

The CHS dataset will then be used to externally validate the model performance to assess transportability. A lack of external validation hampers the implementation of prediction models in routine clinical practice.[40] The prediction model will be applied to each individual in the external validation cohort to give the predicted probabilities of experiencing AF at 6 months. Prediction performance will be quantified by calculating the AUROC, Brier score, and by using calibration plots, and the same aforementioned clinical utility and subgroup analysis will be conducted. Performance of the prediction model will be compared with the CHA_2DS_2-VASc, C_2HEST scores.

<u>Prediction model calculator</u>

For peer review only

The full models are developed to take advantage of rich longitudinal community-based EHRs present in many high income countries. However there are other geographies (low-lower middle income countries) and care setting (emergency care, secondary care clinics) where searching for AF may be desired and an easy-to-use, simple model is preferable. From the derived prediction model, we will generate a parsimonious model based on factors with clinical rationale to predict new-onset AF over a 6 months time horizon.[10] The is will based upon the same core prinicples as detail above, but use logistic regression to ensure transparency in how prediction results are calculated. We will aim to develop a user-friendly version of a model that may be applied as a calculator in a clinical and public setting, yet have good model performance indices.

<u>Software</u>

All analysis will be conducted through R.

For peer review only

ETHICS AND DISSEMINATION

The study has been approved by CPRD (ref no: 19_076). Those handling data have completed University of Leeds information security training. All analyses will be conducted in concordance with the CPRD study dataset agreement between the Secretary of State for Health and Social Care and the University of Leeds.

The Clalit Health Services (CHS) Community Helsinki Committee and the CHS Data Utilization Committee approved the study. The study was exempt from the requirement to obtain informed consent.

The study has been registered at clinical trials.gov (NCT05837364). The study is informed by the Prognosis Research Strategy (PROGRESS) framework and recommendations.[40] The subsequent research papers will be submitted for publication in a peer-reviewed journal and will be written following TRIPOD: *transparent reporting of a multivariable prediction model for individual prognosis or diagnosis* and RECORD: *reporting of studies conducted using observational routinely-collected health data* guidelines,[41 42] as well as the CODE-EHR best-practice framework for using structured electronic healthcare records in clinical research.[43]

If the model shows better prediction performance than previous models and evidence for clinical utility in analysis, it could be made readily available through EHR platforms. The model will be designed to be amenable to in-situ updating with new information so that prediction of an individual's AF risk is updated contemporaneously. If the parsimonious model shows good prediction performance, the user friendly version could be accessible through the internet. Future research would be needed to assess the clinical impact of this risk model. At the point when utilisation in clinical practice is possible the applicable regulation on medicine

For peer review only

devices will be adhered to.[44] When in clinical use, the model itself could also be reviewed and updated by a pre-specified expert consensus group on an annual basis after incorporating evidence from post-service utilization and the curation of more data.

For peer review only

CONCLUSIONS

Atrial fibrillation is a common clinical problem with important clinical sequelae that extend beyond stroke. A prediction model that may identify in a community-based EHR which individuals will develop AF could enable targeted screening. This British Heart Foundation funded study is designed to fill a knowledge gap and enable the leveraging of EHRs to provide risk prediction and targeted AF screening. By understanding if individuals identified as higher risk of new onset AF are also at elevated risk of other cardio-renal-metabolic diseases, this study may demonstrate the opportunity to deliver a more comprehensive clinical approach to improve patient outcomes from AF screening.

For peer review only

Authors' contributions

RN, JW and CPG conceived the concept and planned the analysis. RN wrote the first draft, with contributions from all authors. All authors (RN, JW, CC, DH, RA, DZ, MH, TRB, LR, CPG) approved the final version and jointly take responsibility for the decision to submit the manuscript to be considered for publication.

Funding statement

RN is supported by the British Heart Foundation Clinical Research Training Fellowship (FS/20/12/34789). JW is supported by Barts Charity (MGU0504). The analysis in Clalit HealtH Services is funded by Israel Science Foundation Precision Membership Partnership (Grant #3543/21).

Competing Interests

None declared

For peer review only

REFERENCES

1. Wu J, Nadarajah R, Nakao YM, et al. Temporal trends and patterns in atrial fibrillation incidence: A population-based study of 3· 4 million individuals. *The Lancet Regional Health-Europe* 2022:100386.
2. Svennberg E, Engdahl J, Al-Khalili F, et al. Mass screening for untreated atrial fibrillation: the STROKESTOP study. *Circulation* 2015;131(25):2176-84.
3. Gladstone DJ, Sharma M, Spence JD. Cryptogenic stroke and atrial fibrillation. *The New England journal of medicine* 2014;371(13):1260-60.
4. Ruff CT, Giugliano RP, Braunwald E, et al. Comparison of the efficacy and safety of new oral anticoagulants with warfarin in patients with atrial fibrillation: a meta-analysis of randomised trials. *Lancet* 2014;383(9921):955-62. doi: 10.1016/S0140-6736(13)62343-0 [published Online First: 2013/12/10]
5. Hindricks G, Potpara T, Dagres N, et al. 2020 ESC Guidelines for the diagnosis and management of atrial fibrillation developed in collaboration with the European Association of Cardio-Thoracic Surgery (EACTS). *Eur Heart J* 2020 doi: 10.1093/eurheartj/ehaa612 [published Online First: 2020/08/30]
6. Ruff CT, Giugliano RP, Braunwald E, et al. Comparison of the efficacy and safety of new oral anticoagulants with warfarin in patients with atrial fibrillation: a meta-analysis of randomised trials. *The Lancet* 2014;383(9921):955-62.
7. Kirchhof P, Camm AJ, Goette A, et al. Early rhythm-control therapy in patients with atrial fibrillation. *N Engl J Med* 2020;383(14):1305-16.
8. NHS. Cardiovascular disease 2019 [updated 21 August 2019. Available from: https://www.longtermplan.nhs.uk/areas-of-work/cardiovascular-disease/.
9. Hindricks G, Potpara T, Dagres N, et al. 2020 ESC Guidelines for the diagnosis and management of atrial fibrillation developed in collaboration with the European Association for Cardio-Thoracic Surgery (EACTS) The Task Force for the diagnosis and management of atrial fibrillation of the European Society of Cardiology (ESC) Developed with the special contribution of the European Heart Rhythm Association (EHRA) of the ESC. *Eur Heart J* 2021;42(5):373-498.
10. Hindricks G, Potpara T, Dagres N, et al. 2020 ESC Guidelines for the diagnosis and management of atrial fibrillation developed in collaboration with the European Association for Cardio-Thoracic Surgery (EACTS) The Task Force for the diagnosis and management of atrial fibrillation of the European Society of Cardiology (ESC) Developed with the special contribution of the European Heart Rhythm Association (EHRA) of the ESC. 2021;42(5):373-498.
11. Odutayo A, Wong CX, Hsiao AJ, et al. Atrial fibrillation and risks of cardiovascular disease, renal disease, and death: systematic review and meta-analysis. *BMJ* 2016;354
12. Gladstone DJ, Wachter R, Schmalstieg-Bahr K, et al. Screening for atrial fibrillation in the older population: a randomized clinical trial. *JAMA cardiology* 2021;6(5):558-67.
13. Halcox JP, Wareham K, Cardew A, et al. Assessment of remote heart rhythm sampling using the AliveCor heart monitor to screen for atrial fibrillation: the REHEARSE-AF study. *Circulation* 2017;136(19):1784-94.
14. Steinhubl SR, Waalen J, Edwards AM, et al. Effect of a home-based wearable continuous ECG monitoring patch on detection of undiagnosed atrial fibrillation: the mSToPS randomized clinical trial. *JAMA* 2018;320(2):146-55.

For peer review only

15. Kemp Gudmundsdottir K, Fredriksson T, Svennberg E, et al. Stepwise mass screening for atrial fibrillation using N-terminal B-type natriuretic peptide: the STROKESTOP II study. *EP Europace* 2020;22(1):24-32.
16. Svennberg E, Friberg L, Frykman V, et al. Clinical outcomes in systematic screening for atrial fibrillation (STROKESTOP): a multicentre, parallel group, unmasked, randomised controlled trial. *Lancet* 2021;398(10310):1498-506. doi: 10.1016/S0140-6736(21)01637-8 [published Online First: 2021/09/02]
17. Herrett E, Gallagher AM, Bhaskaran K, et al. Data resource profile: clinical practice research datalink (CPRD). *Int J Epidemiol* 2015;44(3):827-36.
18. Himmelreich JC, Lucassen WA, Harskamp RE, et al. CHARGE-AF in a national routine primary care electronic health records database in the Netherlands: validation for 5-year risk of atrial fibrillation and implications for patient selection in atrial fibrillation screening. 2021;8(1):e001459.
19. Moons KG, Kengne AP, Woodward M, et al. Risk prediction models: I. Development, internal validation, and assessing the incremental value of a new (bio) marker. *Heart* 2012;98(9):683-90.
20. Nadarajah R, Alsaeed E, Hurdus B, et al. Prediction of incident atrial fibrillation in community-based electronic health records: a systematic review with meta-analysis. *Heart* 2021
21. Herrett E, Thomas SL, Schoonen WM, et al. Validation and validity of diagnoses in the General Practice Research Database: a systematic review. *Br J Clin Pharmacol* 2010;69(1):4-14.
22. Chisholm J. The Read clinical classification. *BMJ: British Medical Journal* 1990;300(6732):1092.
23. Hill NR, Ayoubkhani D, McEwan P, et al. Predicting atrial fibrillation in primary care using machine learning. *PLoS One* 2019;14(11):e0224582.
24. Arbel R, Hammerman A, Sergienko R, et al. BNT162b2 vaccine booster and mortality due to Covid-19. *N Engl J Med* 2021;385(26):2413-20.
25. Arbel R, Sergienko R, Friger M, et al. Effectiveness of a second BNT162b2 booster vaccine against hospitalization and death from COVID-19 in adults aged over 60 years. *Nat Med* 2022;28(7):1486-90.
26. Arbel R, Wolff Sagy Y, Hoshen M, et al. Nirmatrelvir use and severe Covid-19 outcomes during the Omicron surge. *N Engl J Med* 2022;387(9):790-98.
27. Hammerman A, Sergienko R, Friger M, et al. Effectiveness of the BNT162b2 vaccine after recovery from Covid-19. *N Engl J Med* 2022;386(13):1221-29.
28. Riley RD, Snell KI, Ensor J, et al. Minimum sample size for developing a multivariable prediction model: PART II-binary and time-to-event outcomes. *Stat Med* 2019;38(7):1276-96.
29. Cowan JC, Wu J, Hall M, et al. A 10 year study of hospitalized atrial fibrillation-related stroke in England and its association with uptake of oral anticoagulation. *Eur Heart J* 2018;39(32):2975-83.
30. Wu J, Alsaeed ES, Barrett J, et al. Prescription of oral anticoagulants and antiplatelets for stroke prophylaxis in atrial fibrillation: nationwide time series ecological analysis. *EP Europace* 2020;22(9):1311-19.
31. Himmelreich JC, Veelers L, Lucassen WA, et al. Prediction models for atrial fibrillation applicable in the community: a systematic review and meta-analysis. *EP Europace* 2020;22(5):684-94.

For peer review only

32. Routen A, Akbari A, Banerjee A, et al. Strategies to record and use ethnicity information in routine health data. *Nat Med* 2022:1-4.
33. Groenwold RH. Informative missingness in electronic health record systems: the curse of knowing. *Diagnostic and prognostic research* 2020;4(1):1-6.
34. Breiman L. Random forests. *Machine learning* 2001;45(1):5-32.
35. Attia ZI, Noseworthy PA, Lopez-Jimenez F, et al. An artificial intelligence-enabled ECG algorithm for the identification of patients with atrial fibrillation during sinus rhythm: a retrospective analysis of outcome prediction. *The Lancet* 2019;394(10201):861-67.
36. Sakamoto Y, Ishiguro M, Kitagawa G. Akaike information criterion statistics. *Dordrecht, The Netherlands: D Reidel* 1986;81(10.5555):26853.
37. Szymanski T, Ashton R, Sekelj S, et al. Budget impact analysis of a machine learning algorithm to predict high risk of atrial fibrillation among primary care patients. *EP Europace* 2022
38. McDonagh TA, Metra M, Adamo M, et al. 2021 ESC Guidelines for the diagnosis and treatment of acute and chronic heart failure: Developed by the Task Force for the diagnosis and treatment of acute and chronic heart failure of the European Society of Cardiology (ESC) With the special contribution of the Heart Failure Association (HFA) of the ESC. *Eur Heart J* 2021;42(36):3599-726.
39. Vahanian A, Beyersdorf F, Praz F, et al. 2021 ESC/EACTS Guidelines for the management of valvular heart disease: developed by the Task Force for the management of valvular heart disease of the European Society of Cardiology (ESC) and the European Association for Cardio-Thoracic Surgery (EACTS). *Eur Heart J* 2022;43(7):561-632.
40. Steyerberg EW, Moons KG, van der Windt DA, et al. Prognosis Research Strategy (PROGRESS) 3: prognostic model research. *PLoS Med* 2013;10(2):e1001381.
41. Collins GS, Reitsma JB, Altman DG, et al. Transparent Reporting of a Multivariable Prediction Model for Individual Prognosis or Diagnosis (TRIPOD) The TRIPOD Statement. *Circulation* 2015;131(2):211-19.
42. Nicholls SG, Quach P, von Elm E, et al. The reporting of studies conducted using observational routinely-collected health data (RECORD) statement: methods for arriving at consensus and developing reporting guidelines. *PLoS One* 2015;10(5):e0125620.
43. Kotecha D, Asselbergs FW, Achenbach S, et al. CODE-EHR best practice framework for the use of structured electronic healthcare records in clinical research. *BMJ* 2022;378
44. Kramer DB, Xu S, Kesselheim AS. Regulation of medical devices in the United States and European Union. The Ethical Challenges of Emerging Medical Technologies: Taylor and Francis 2020:41-49.

For peer review only

Patient consent for publication

Not required

Ethics Approval

Permissions for the CPRD-GOLD and CPRD-AURUM datasets were obtained from CPRD (ref no: 19_076). The study was approved by CPRD ethical approval committee. The Clalit Health Services (CHS) Community Helsinki Committee and the CHS Data Utilization Committee approved the study. The study was exempt from the requirement to obtain informed consent.

Word Count

3911

Figure Legend

Figure 1, A schematic representation comparing current AF screening approaches, which focus on stroke prevention, with a broader approach to AF screening that considers that individuals eligible for AF screening will be at risk of multiple outcomes beyond stroke.

Figure 2. A schematic representation of a multivariable logistic regression model or random forest model using data from electronic health records to provide risk prediction for incident AF.

For peer review only

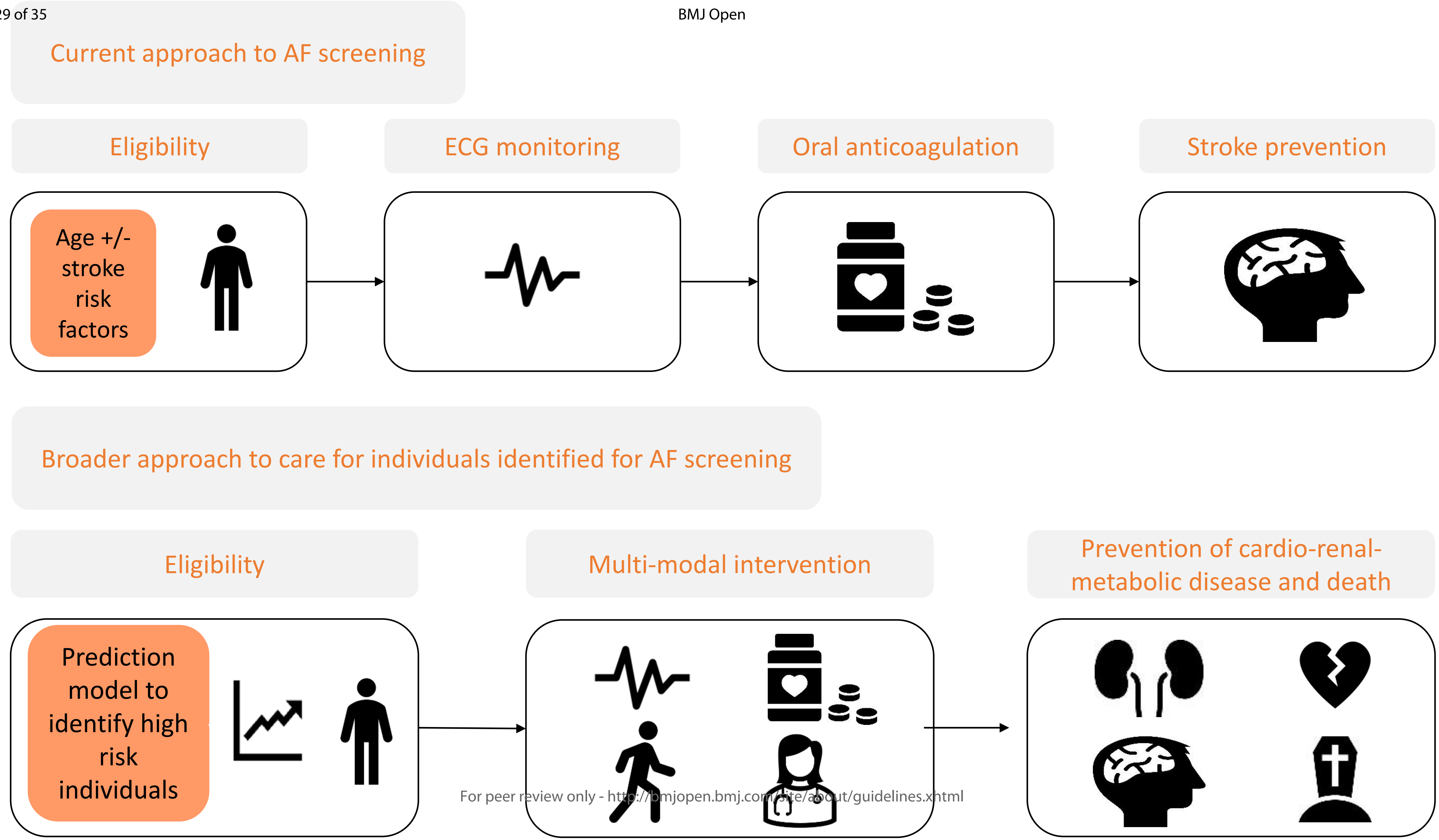

For peer review only - http://bmjopen.bmj.com/site/about/guidelines.xhtml

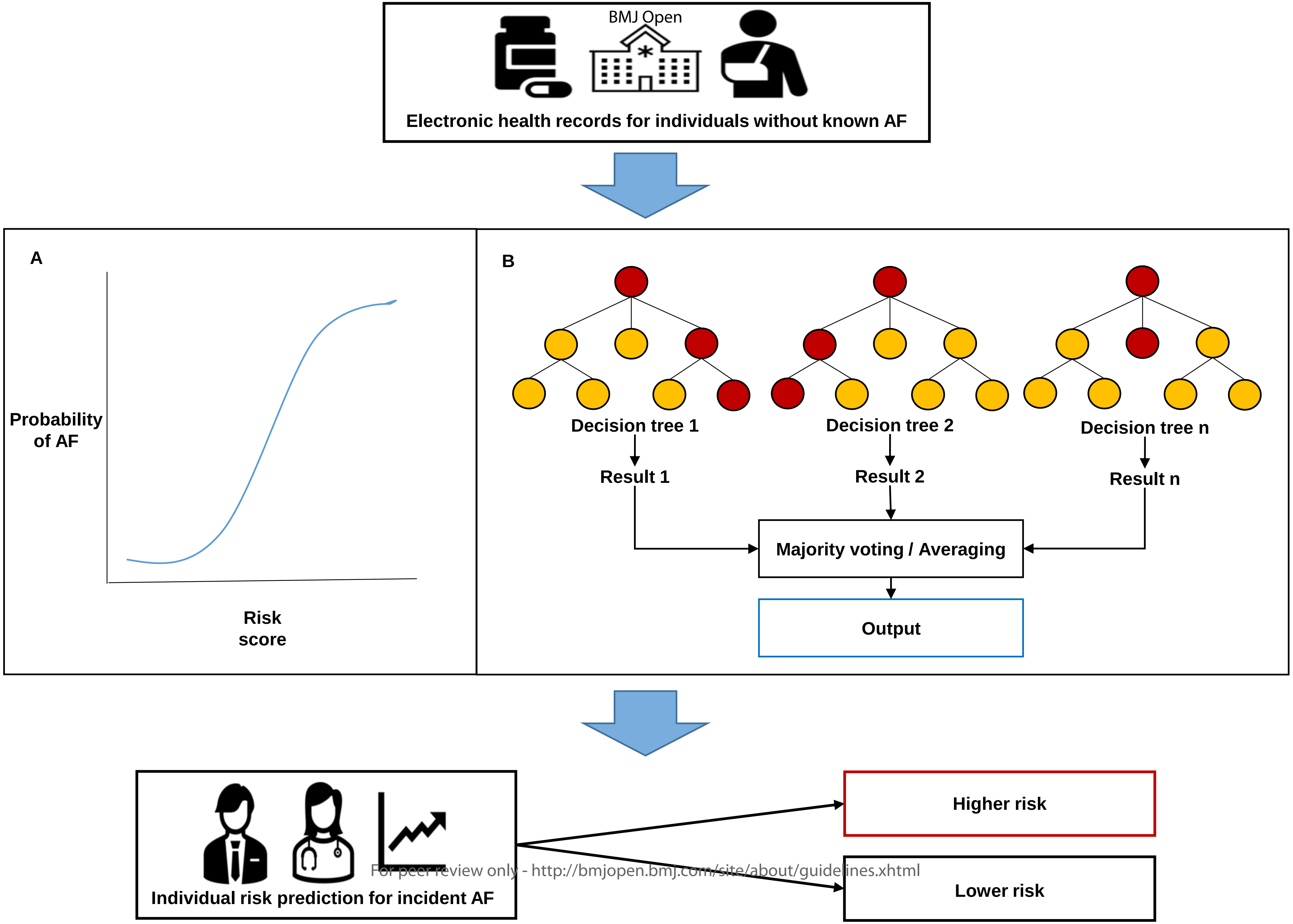

For peer review only - http://bmjopen.bmj.com/site/about/guidelines.xhtml

Supplementary Appendix

Risk of atrial fibrillation and association with other diseases: protocol of the derivation and international external validation of a prediction model using nationwide population-based electronic health records

Ramesh Nadarajah, Jianhua Wu, Ronen Arbel, Moti Haim, Doron Zahger, Talish Razi Benita, Lior Rokach, Campbell Cowan, Chris P Gale

Supplementary Table 1. Baseline demographic and comorbidity variables used in algorithms tested for predicting incident AF in community-based electronic health records 2

Supplementary Table 2. Definition of disease categories for causes of deaths 4

Supplementary Figure 1. Design process leading to selection of non-AF outcomes to assess for association with predicted AF risk 5

For peer review only

For peer review only - http://bmjopen.bmj.com/site/about/guidelines.xhtml

Supplementary Table 1. Baseline demographic and comorbidity variables used in algorithms tested for predicting incident AF in community-based electronic health records

Algorithm	Demographics	Comorbidities
$CHADS_2$	Age	Hypertension, CHF, diabetes mellitus, CVA
CHA_2DS_2-VASc	Age, sex	Hypertension, CHF, stroke/TIA/thromboembolism, vascular disease
CHARGE-AF	Age, race, smoking status	Anti-hypertensive medication, MI, CHF, DM
C_2HEST	Age	Hypertension, ischaemic heart disease, CHF, COPD, thyroid disease
HATCH	Age	Hypertension, CHF, stroke/TIA, COPD
InGef	Age, sex	Anti-hypertension medication, heart failure medication, chronic kidney disease, disorderd of lipoprotein metabolism and other lipidaemias, pulmonary heart diseases cardiac arrhythmias, other cerebrovascular disease, diverticular disease of intestine, dorsalgia, breathing abnormalities
MHS	Age, sex	Anti-hypertensive medication, MI, CHF, peripheral vascular disease, inflammatory disease in a female, COPD
NHIRD	Age (years), age group, sex	Hypertension, CHF, COPD, rheumatological disease, dyslipidaemia, DM, CVA or TIA, sleep disorder, cancer, hyperthyroidism, vascular disease, gout, CKD or ESRD, anaemia
NHIS-NSC*	Age, sex, smoking (pack-year), alcohol	Hypertension, CHF, MI, vascular disease, stroke/TIA, COPD
Pfizer-AI	Age, sex, race, smoking status	Hypertension, anti-hypertensive medication, CHF, congenital heart disease, MI, LVH, type 1 DM, type 2 DM
Taiwan AF	Age, sex, alcohol excess	Hypertension, CHF, IHD, ESRD

AF, Atrial Fibrillation; $CHADS_2$, Congestive heart failure, Hypertension, Age >75, Diabetes mellitus, prior Stroke or transient ischemic attack [2 points]; CHA_2DS_2-VASc, Congestive heart failure, Hypertension, Age >75 [2 points], Stroke/transient ischemic attack/thromboembolism [2 points]; CHARGE-AF, Cohorts for Heart and Aging Research in Genomic Epidemiology; C_2HEST, Coronary artery disease / Chronic obstructive pulmonary disease [1 point each], Hypertension, Elderly (Age ≥75, 2 points), Systolic heart failure, Thyroid disease (hyperthyroidism); CHF, chronic heart failure; CKD, chronic kidney disease; COPD, chronic obstructive pulmonary disease; CPRD, Clinical Practice Research Datalink; CVA, cerebrovascular accident; DM, diabetes mellitus; ESRD, end-stage renal disease; HATCH, Hypertension, Age, stroke or Transient ischemic attack, Chronic obstructive pulmonary disease, Heart failure; IHD, ischaemic heart disease; LVH, left ventricular hypertrophy; MHS, Maccabi Healthcare Services; MI, myocardial infarction; NHIRD, National Health Insurance Research Database; NHIS-HEALS, National Health Insurance Service - Health screening Cohort; NHIS-NSC, National Health Insurance Service-based National Sample Cohort; TIA, transient ischaemic attack.

For peer review only - http://bmjopen.bmj.com/site/about/guidelines.xhtml

* In Kim 2020 prediction model development using machine learning was completed both with and without the predictor $PM_{2.5}$ - which is fine particular matter air pollution. In this analysis we have only included the model without $PM_{2.5}$ as it is judged not to be a predictor that would be routinely available in primary care or population EHR.

For peer review only - http://bmjopen.bmj.com/site/about/guidelines.xhtml

Supplementary Table 2. Definition of disease categories for causes of deaths

Causes of death	Code
**Cardiovascular disorders**	ICD chapter 'Diseases of the circulatory system' (code range: I00–I99), excluding codes relating to infections or cerebrovascular disease.
**Cerebrovascular disorders**	ICD chapter 'Diseases of the circulatory system' (I60-I69)
**Neoplasms**	ICD chapter 'Neoplasms' (C00–D48).
**Infections**	Infectious and parasitic diseases, respiratory infections, urinary tract infections, and cellulitis, as defined by individual codes as Conrad et al.
**Chronic respiratory diseases**	Individual codes Conrad et al.
**Digestive diseases**	ICD chapter 'Diseases of the digestive system' (K00–K93), excepting selected codes categorized as infections.
**Mental and neurological disorders**	ICD chapter 'Mental and behavioral disorders' (F00–F99) and ICD chapter 'Diseases of the nervous system' (G00–G99)
**Injuries**	ICD chapters 'Injury, poisoning and certain other consequences of external causes' (S00–T98) and 'External causes of morbidity and mortality' (V01–Y98)
**Kidney diseases**	ICD sub-chapters 'Renal failure' (N17-N19), 'Glomerular diseases' (N00-N08), 'Renal tubulo-interstitial diseases' (N10-N16), 'Other disorders of kidney and ureter' (N25-N29)

To categorise cause of death as infections or chronic respiratory diseases we used the same codelists as Conrad N, Judge A, Canoy D, et al. Temporal trends and patterns in mortality after incident heart failure: a longitudinal analysis of 86 000 individuals. *JAMA cardiology* 2019;4(11):1102-11

For peer review only - http://bmjopen.bmj.com/site/about/guidelines.xhtml

Supplementary Figure 1. Design process leading to selection of non-AF outcomes to assess for association with predicted AF risk

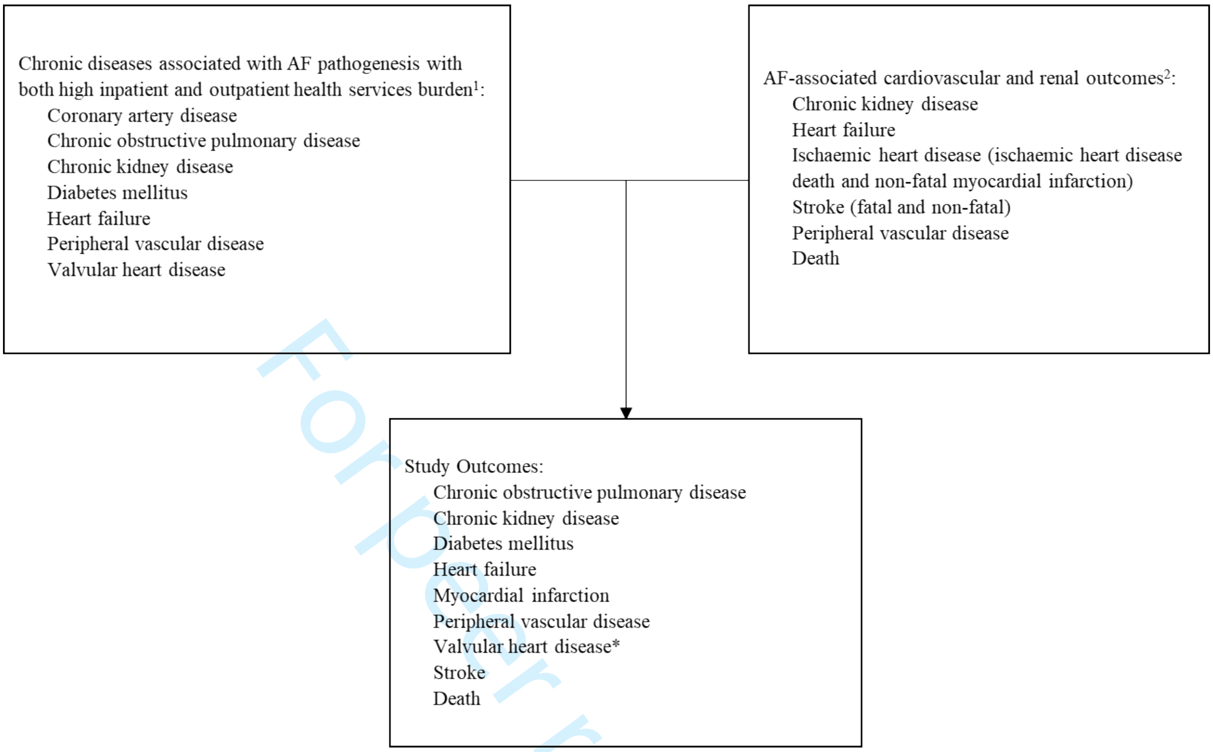

[1] Hindricks G, Potpara T, Dagres N, et al. 2020 ESC Guidelines for the diagnosis and management of atrial fibrillation developed in collaboration with the European Association for Cardio-Thoracic Surgery (EACTS) The Task Force for the diagnosis and management of atrial fibrillation of the European Society of Cardiology (ESC) Developed with the special contribution of the European Heart Rhythm Association (EHRA) of the ESC. 2021;42(5):373-498.

[2] Odutayo A, Wong CX, Hsiao AJ, et al. Atrial fibrillation and risks of cardiovascular disease, renal disease, and death: systematic review and meta-analysis. *BMJ* 2016;354

* Aortic stenosis was further specified in addition to valvular heart disease given the increasing availability and randomised controlled trial evidence for earlier treatment, and increasing therapeutic options across operative risk profiles (Vahanian A, Beyersdorf F, Praz F, et al. 2021 ESC/EACTS Guidelines for the management of valvular heart disease: developed by the Task Force for the management of valvular heart disease of the European Society of Cardiology (ESC) and the European Association for Cardio-Thoracic Surgery (EACTS). *Eur Heart J* 2022;43(7):561-632.)

For peer review only

For peer review only - http://bmjopen.bmj.com/site/about/guidelines.xhtml

BMJ Open

Risk of atrial fibrillation and association with other diseases: protocol of the derivation and international external validation of a prediction model using nationwide population-based electronic health records

Journal:	*BMJ Open*
Manuscript ID	bmjopen-2023-075196.R1
Article Type:	Protocol
Date Submitted by the Author:	08-Sep-2023
Complete List of Authors:	Nadarajah, Ramesh; University of Leeds, Leeds Institute of Data Analytics; Leeds Teaching Hospitals NHS Trust, Department of Cardiology<br>Wu, Jianhua; University of Leeds, Leeds Institute for Data Analytics; Queen Mary University of London, Wolfson Institute of Population Health<br>Arbel, Ronen; Ben-Gurion University of the Negev, Health Systems Management; Sapir College,<br>Haim, Moti; Soroka University Medical Center, Department of Cardiology; Ben-Gurion University of the Negev<br>Zahger , Doron ; Soroka University Medical Center<br>Benita, Talish Razi; Clalit Health Services; Ben-Gurion University of the Negev<br>Rokach, Lior; Ben-Gurion University of the Negev, Department of Information Systems and Software Engineering<br>Cowan, Campbell; Leeds Teaching Hospitals NHS Trust, Department of Cardiology<br>Gale, Chris; University of Leeds
**Primary Subject Heading**:	Cardiovascular medicine
Secondary Subject Heading:	General practice / Family practice, Public health
Keywords:	Electronic Health Records, Pacing & electrophysiology < CARDIOLOGY, PREVENTIVE MEDICINE, Primary Care < Primary Health Care

SCHOLARONE™
Manuscripts

Title:

Risk of atrial fibrillation and association with other diseases: protocol of the derivation and international external validation of a prediction model using nationwide population-based electronic health records

Publication type: Study Protocol

Target journal: BMJ Open

Authors:

Ramesh Nadarajah[1,2,3], Jianhua Wu[4,5], Ronen Arbel[6,7], Moti Haim[8,9], Doron Zahger[10,11], Talish Razi Benita[6,9], Lior Rokach[10], Campbell Cowan[3], Chris P Gale[1,2,3]

Affiliations:

[1] Leeds Institute for Cardiovascular and Metabolic Medicine, University of Leeds, UK

[2] Leeds Institute of Data Analytics, University of Leeds, UK

[3] Department of Cardiology, Leeds Teaching Hospitals NHS Trust, Leeds, UK

[4] Wolfson Institute of Population Health, Queen Mary, University of London, UK

[5] School of Dentistry, University of Leeds, Leeds, UK

[6] Community Medical Services Division, Clalit Health Services, Tel Aviv, Israel

[7] Maximizing Health Outcomes Research Lab, Sapir College, Sderot, Israel

[8] Department of Cardiology, Soroka University Medical Center, Beer Sheva, Israel

[9] Faculty of Health Sciences, Ben Gurion University of the Negev, Beer Sheva, Israel

[10] Department of Information Systems and Software Engineering, Ben Gurion University of the Negev, Beer-Sheva, Israel

For peer review only

Correspondence:

Ramesh Nadarajah

British Heart Foundation Clinical Research Fellow

Leeds Institute for Cardiovascular and Metabolic Medicine

University of Leeds

6 Clarendon Way

Leeds, UK

LS2 9DA

Tel +44 (0) 113 343 3241

Email r.nadarajah@leeds.ac.uk

Twitter @Dr_R_Nadarajah

Word Count

3999

Keywords

Atrial Fibrillation

Prediction

Screening

Community

Electronic health records

Cardiometabolic

Outcomes

For peer review only

Risk

Population

For peer review only

Abstract

Introduction

Atrial fibrillation (AF) is a major public health issue and there is rationale for the early diagnosis of AF, before the first complication occurs. Previous AF screening research is limited by low yields of new cases and strokes prevented in the screened populations. For AF screening to be clinically and cost-effective, the efficieny of identification of newly diagnosed AF needs to be improved and the intervention offered may have to extend beyond oral anticoagulation for stroke prophylaxis. Previous prediction models for incident AF have been limited by their data sources and methodologies.

Methods and analysis

We will investigate the application of Random Forest and multivariable logistic regression to predict incident AF within a 6 months prediction horizon, that is a time-window consistent with conducting investigation for AF. The Clinical Practice Research Datalink (CPRD)-GOLD dataset will be used for derivation, and the Clalit Health Services dataset will be used for international external geographical validation. Analyses will include metrics of prediction performance and clinical utility. We will create Kaplan-Meier plots for individuals identified as higher and lower predicted risk of AF and derive the cumulative incidence rate for non-AF cardio-renal-metabolic diseases and death over the longer term to establish how predicted AF risk is associated with a range of new non-AF disease states.

Ethics and dissemination

Permission for CPRD-GOLD was obtained from CPRD (ref no: 19_076). The CPRD ethical approval committee approved the study. CHS Helsinki committee approval 21-0169 and data

For peer review only

utilization committee approval 901. The results will be submitted as a research paper for publication to a peer-reviewed journal and presented at peer-reviewed conferences.

Trial registration details

A systematic review to guide the overall project was registered on PROSPERO (registration number CRD42021245093). The study was registered on Clinical Trials.gov (NCT05837364).

For peer review only

Strengths and limitations of the study

- Large and nationwide datasets representative of the community-dwelling populations in two countries.
- Predicting risk of incident AF in the short-term may be more useful to screening than longer prediction horizons
- Quantification of the strength of association between predicted AF risk and other diseases may uncover other opportunities that could be actioned during AF screening beyond stroke prophylaxis.
- A calculator created from a parsimonious model may enhance the usability of the model in the real world and in contexts where electronic health records are unavailable or incomplete.
- It is estimated that more than a quarter of individuals living with AF are not diagnosed during routine care, which may mean that the performance of the prediction model may vary in a screening setting.

For peer review only

INTRODUCTION

Atrial fibrillation (AF) is the most common sustained cardiac arrhythmia. Over the last 20 years the number of new cases of AF diagnosed each year has risen by 72%, and now surpasses the four most common causes of cancer combined.(1) Moreover, it is estimated that up to 35% of disease burden remains undiagnosed,(2) and 15% of strokes occur in the context of undiagnosed AF.(3)

Oral anticoagulants can reduce the risk of stroke by up to two thirds in those with AF at higher risk of stroke,(4) and international guidelines recommend their use in patients with AF at elevated thromboembolic risk.(5) Early detection of AF may permit the initiation of oral anticoagulation to reduce embolic stroke risk,(6) and early antiarrhythmic therapy to reduce the risk of death and stroke.(7) Accordingly early AF detection is a key cardiovascular priority in the UK NHS Long Term Plan,(8) and the European Society of Cardiology recommends opportunistic screening by pulse palpation or electrocardiogram (ECG) rhythm strip in persons aged ≥65 years and systematic ECG screening in those aged ≥75 years.(9)

Furthermore, AF frequently develops due to, and in parallel with, other cardiovascular, renal and metabolic conditions,(10) and individuals with AF are at an increased risk of major cardiovascular events in excess of stroke including ischemic heart disease, heart failure, chronic kidney disease, peripheral vascular disease and death.(11) Thus, AF screening, with or without AF diagnosis, may be a key opportunity for holistic management of cardiometabolic risk factors and unhealthy lifestyle behaviours to reduce an individual's risk of later adverse events beyond that of stroke prophylaxis alone.

For peer review only

Several randomised clinical trials (RCT) have shown that serial or continuous non-invasive electrocardiogram (ECG) monitoring in older people with stroke risk factors / elevated N-terminal pro B-type natriutretic peptide (NT-proBNP), leads to a higher detection rate of previously undiagnosed AF compared with routine standard of care, though yields remain relatively low (3.0%-4.4%).(12-15) The STROKESTOP RCT, where AF screening was offered to individuals aged 75 and 76 years without exclsions, achieved only a 3% yield of new AF cases with a modest benefit in a composite outcome of ischaemic or haemorrhagic stroke, systemic embolism, bleeding leading to hospitalisation and all-cause death; and not for each of ischaemic stroke, haemorrhagic stroke, or hospitalisation for major bleeding.(16) Accordingly, for AF screening to be effective the yield of newly diagnosed AF amongst participants needs to be improved and the intervention offered may have to extend beyond only oral anticoagulation for stroke prophylaxis (Figure 1).

A large proportion of the population are registered in primary care with a routinely-collected electronic health record (EHR).(17 18) A prediction model that utilises data available in the community to calculate AF risk could discriminate patients into risk categories, with screening offered only to higher risk individuals,(19) enabling scalable and efficient targeted AF screening. To date, several multivariable prediction models have been created or tested for prediction of incident AF in community-based electronic health records, but are of limited clinical utility for AF screening on account of moderate discriminative performance, long prediction horizons and limited scalability due to missing data.(20) None have yet reached widespread clinical practice. Moreover, reports of prediction models have yet to quantify the association between AF risk and new disease states outside that of AF and stroke.

For peer review only

Research aim

The aims of this study are to:

1) Develop a model for predicting short-term AF risk from data routinely available in community-based EHRs.
2) Quantify the association of predicted AF risk with a range of non-AF diseases.
3) Externally validate the prediction model in an international context to assess transportability.
4) Produce a calculator derived from a parsimonious prediction model.

For peer review only

METHODS AND ANALYSIS

Data sources and permissions

The derivation dataset will be the Clinical Practice Research Datalink-GOLD (CPRD-GOLD) dataset. This is an ongoing primary care database, established in 1987, that comprises anonymised medical records and prescribing data contributed by general practices using Vision® software. It contains data for approximately 17.5 million patients, with 30% of contributing practices in England, and represents the United Kingdom (UK) population in terms of age, sex and ethnicity.(17) In order to contribute to the database, general practices and other health centres must meet prespecified standards for research-quality data ('up-to-standard').(17 21)

Recorded information includes patients' demography, clinical symptoms, signs, investigations, diagnoses, prescriptions, referrals, behavioural factors and test results entered by clinicians and other practice staff. All clinical information is coded using Read Codes.(22) Extracted patients will have patient-level data linked to Hospital Episode Statistics (HES) Admitted Patient Care (APC) and Office for National statistics (ONS) Death Registration. The CPRD dataset has been used to develop or validate a range of risk prediction models.(23)

The extracted dataset, including linked data, comprises all patients for the period between 2nd January 1998 and 30th November 2018 from the snapshot of CPRD-GOLD in October 2019. Over this study period, the CPRD-GOLD dataset comprises approximately 2 million patients eligible for data linkage at an up-to-standard practice, with over 200,000 patients having a record of AF during follow-up.

For peer review only

To ascertain whether the prediction model is transportable to geographies outside of the UK, we will externally validate its performance in the Clalit Health Services database in Israel. As a result of the National Health Insurance Law, Israeli citizens are required to enroll in 1 of 4 payer-provider health funds and receive free basic health care. Clalit Health Services (CHS) provides health insurance coverage to 4.8 million insured members, and about two thirds of the population aged >65 years. CHS is recooginzed globally as the primary source of evaluation of Covid-19 vaccinations and therapies.(24-27) All clinical information is coded in International Classifications of Diseases, Ninth Revision (ICD-9). Receipt of vital status from the Ministry of the Interior ensures 100% follow-up of mortality. We will include participants insured by Clalit with continuous membership for at least 1 year before 01/01/2019: 2,159,663 patients with 4,330 of them having a new icident of AF (Atrial fibrillation and/or atrial flutter) in the first half of 2019.

Patient and Public Involvement

The Arrhythmia Alliance and AF association provided input on the scientific advisory board for this research programme, and our patient and public involvement group have given input to reporting and dissemination plans of the research.

Inclusion and exclusion criteria

The study population for derivration and internal validation will comprise all available patients in CPRD-GOLD eligible for data linkage and with at least 1-year follow-up in the period between 2nd January 1998 and 30th November 2018. For the external validation the study population will comprise participants insured by CHS, including those with continuous membership for at least 1 year, before 01/01/2019 . Patients will be excluded if they were ≤30 years of age, or diagnosed with AF or atrial flutter (AFl) at the point of study entry, registered

For peer review only

for less than 1 year or, in CPRD, ineligible for data linkage. Patients younger than 30 years of age are not included in the cohort for AF prediction because the incidence of AF over even a 10-year horizon is very low in this group.(1)

Prediction model outcome ascertainment

The outcome of interest is first diagnosed AF or AFl after baseline. Baseline is taken in the CPRD-GOLD dataset as the first entry of the patient into the dataset. We have included AFl as an outcome since it has similar clinical relevance, including thromboembolic risk and anticoagulation guidelines, as AF.(5) These will be identified using Read codes in CPRD dataset. For HES APC events and underlying cause of death variable in the ONS Death Registration data file, ICD-10 codes will be used. For CHS events will be identified using ICD-9 codes. It should be noted that a report has estimated that 305 262 individuals in the UK have undiagnosed AF,(28) and so incidence of AF within the study may be underestimated as there will be individuals with unrecorded asymptomatic AF.

Sample size

To develop a prognostic prediction model, the required sample size may be determined by three criteria suggested by *Riley et al*.(29) For example, suppose a maximum of 200 parameters will be included in the prediction model and the Cox-Snell generalised R^2 is assumed to be 0.01. A total of 377,996 patients will be required to meet Riley's criterion (i) with global shrinkage factor of 0.95; this sample size also ensures a small absolute difference ($\Delta < 0.05$) in the apparent and adjusted Nagelkerke R^2 (Riley's criterion (ii)) and ensures precise estimate of overall risk with a margin of error < 0.001 (Riley's criterion (iii)). According to the Quality and Outcomes Framework (QOF), the prevalence of AF in England is 1.7%.(30 31) Given an AF prevalence of 1.7%, only 6,425 patients will be expected to develop AF from 377,996

patients. Within the Clalit Health Services database there are 2,159,663 patients. Therefore, the number of patients in the CPRD and Clalit health services datasets with AF will provide sufficient statistical power to develop and validate a prediction model with the predefined precision and accuracy.

Predictor Variables

A systematic review has been conducted to establish predictor variables included in varying combinations by preceding prediction models developed to detect incident AF in community-based EHRs (Supplementary Table 1),(32) and supplemented with a literature search for variables associated with incident AF.

Candidate variables include

1. Sociodemographic variables including age, sex and ethnicity (SocioEconomic Score and population sector will serve as surrogate for ethnicity in CHS)
2. All disease conditions included in the patient's record, including hospitalised diseases and procedures, such as other cardiovascular diseases, diabetes mellitus, chronic lung disease, renal disease, inflammatory disease, cancer, hypothyroidism and surgical procedures.
3. Lifestyle factors including smoking status and alcohol consumption that are coded in structured Read codes.

Predictive factors will be identified using the appropriate codes, with Read codes for diagnoses and lifestyle factors. Code lists for predictors will be used from publications if available, otherwise the CPRD code browser will be used and codes checked by at least two clinicians. The code lists for predictors in CPRD-GOLD will be adapted from CALIBER and HDR UK

For peer review only

repositories or publications. If none are available from these sources then new code lists developed using the OpenCodelists and checked by at least two clinicians. Diagnostic code lists will comprise the primary care coding system (Read codes), to ensure that only information readily available within a primary care EHR could be incorporated within the prediction model. Within CHS, the code lists for predictors will be developed using similar methods based on the medical records and coding of CHS, which also includes a validated chronic diseases registry.

Candidate variable data types are deliberately limited to ensure widespread applicability of the model given the reality of 'missing' data in routinely-collected electronic health records.(18) Observations and laboratory results are not included. Ethnicity information is routinely collected in the UK NHS and so has increasingly high completeness,(33) and we will include an 'ethnicity unrecorded' category where it is unavailable because missingness is considered informative.(34) Ethicity in a UK context does not directly translate to an Israeli context so sociodemographic surrgoates will be used: i) .population sectors- General Jewish, ultra-orthodox Jewish and Arab ii). Socioeconomic score on a scale of 1-10. For diagnoses, if a medical code is present in the patient record (without a preceding time window limitation) then the variable is classified as being present for the patient. If medical codes are absent in a patient record we will assume that the patient does not have that diagnosis, or that the diagnosis was not considered sufficiently important to have been recorded by the GP in case of symptoms.(35) Concordantly, the analytical cohorts are not expected to have missing data for any of the predictor variables. It is possible that diagnoses may be recorded as free text, data to which we do not have access, rather than as diagnostic codes and that this may lead to misclassification of some patients.

For peer review only

Data analysis plan

Data pre-processing

The CPRD-GOLD and Clalit Health Services data will be cleaned and preprocessed for model development, internal validation and external validation. Specifically, for patient features with binary values, 0 and 1 will be mapped to the binary values. Variables with multiple categories (ethnicity) will be split into their component categories, and each given a binary value to indicate the presence or not of the variable for each patient. Continuous variables (age) will be kept as continuous.

Descriptive analysis

Continuous variables will be reported as mean ± standard deviation (SD) and categorical variables as frequencies with corresponding percentages.

Prediction model development

We will compare a machine learning and logistic regression approach to prediction model development for incident AF in CPRD-GOLD. Logistic regression model offers a more manageable approach for implementation, interpretation and training compared to machine learning algorithms, but machine learning methods can better handle non-linearities and interactions among variables and may lead to better discriminative performance.(20)

We will investigate the use of a random forest classifier for AF prediction in the CPRD-GOLD dataset. In our systematic review of AF prediction in EHRs it had the most evidence for use and showed robust performance in different datasets and geographies.(20) Random Forest (RF) is an ensemble technique that combines a large number of decision trees using a bagging approach to improve the overall performance (Figure 2).(36) In brief, the bagging approach

For peer review only

grows multiple classification trees in parallel where each tree gives a classification which are called votes. These votes are then aggregated to provide a more accurate and stable prediction. Furthermore the degree of variation of each feature in a RF classifier for the prediction task can be calculated using the mean decrease in the Gini coefficient, a measure of how each variable contributes to the homogeneity of nodes and leaves in the resulting RF. Showing the importance of variables used in prediction (explainability) is considered important for clinical uptake of prediction models,(37) and a limitation of using deep learning techniques. The RF model will be trained in the training dataset to predict a binary classification of developing AF or not. The model will return the probability between 0 and 1 for developing AF in the training set, corresponding to the predicted probabilities of developing AF.

Preprocessed patient-level data in CPRD-GOLD will be randomly split into an 80:20 ratio to create derivation and internal validation (or training and testing) samples. The split ratio is not a significant factor, given the volume of the sample size. The model parameters and dropout rate, will be chosen through a grid search and 10-fold cross-validation will be used (i.e. 10% of the training data will be randomly selected as the cross-validation set). The multivariable logistic regression model will be developed with backward model selection with Akaike information criterion.(38) The prediction window will be set at 6 months, as this is considered in keeping with the logistical time frames for organising AF investigation at scale.

Internal validation

We will evaluate the model performance using a validation cohort with internal bootstrap validation with 200 samples. The model will be applied to the testing dataset with the same predictor variables. The AUROC will be used to evaluate predictive ability (concordance index) with 95% confidence intervals calculated using the DeLong method.(39) Youden's

For peer review only

index will be established for the outcome measure as a method of empirically identifying the optimal dichotomous cut-off to assess sensitivity, specificity, positive predictive value and negative predictive value. We will calculate the Brier score, a measure of both discrimination and calibration, by taking the mean squared difference between predicted probabilities and the observed outcome. To assess the clinical impact of utilising prediction model as opposed to other risk prediction scores, we will calculate the case reclassification, non-case reclassification, and overall net reclassification index at the risk threshold that equates to the average 6 months incidence rate in the cohort and conduct a decision curve analysis, which assesses across threshold probabilities whether the predictive model would do more benefit than harm. Calibration will be assessed graphically by plotting predicted AF risk against observed AF incidence and quantified using a calibration slope.

The same methods will be employed in subgroups by age (<65 years, ≥65 years, <75 years, ≥75 years), sex (women, men) and ethnicity (White, Black, Asian, others and unspecified) to assess the model's predictive performance across clinically relevant groups.

Performance of the prediction model will be compared with the CHA_2DS_2-VASc and C_2HEST scores. The CHA_2DS_2-VASc score was originally developed to predict stroke risk in individuals with AF, and the C_2HEST score for Asian people without structural heart disease.(20) These algorithms are robust to missing data in routinely-collected primary care EHRs and have been tested for AF risk prediction in European cohorts.(20) Other algorithms that can only be applied to a minority of European primary care EHRs (Pfizer-AI, CHARGE-AF) will not be considered as they cannot be implemented at scale to inform AF screening.(18 28)

For peer review only

Quantification of the association between short-term predicted AF risk and long-term AF and other diseases

We will include all patients randomly assigned to the testing dataset in CPRD-GOLD by the Mersenne twister pseudorandom number generator, categorized as lower or higher predicted AF risk by the developed prediction model at baseline (point of entry to the study). For long-term AF risk we will plot Kaplan-Meier plots for individuals identified as higher and lower predicted risk of AF to assess the event rate for AF censored at 10 years, and calculate the hazard ratio for AF between higher and lower predicted risk of AF using the Cox proportional hazard model with adjustment for the competing risk of death. This will inform us of whether short-term AF risk is also associated with long-term AF risk, and whether an individual who undergoes risk-guided AF screening should be considered for repeated AF screening at a later time point (e.g. 1 or 5-years).

For non-AF disease states we will consider the initial presentation of a cardiovascular, renal, or metabolic disease or death. This is because AF is not a disease in isolation and is known to be associated with high risk of adverse clinical outcomes. To best characterise highly prevalent and morbid diseases, associated with the development or consequence of AF and that may be appropriate for prevention or targeted diagnostic pathways subsequent to AF screening (Supplementary Figure 1),(10) we will individually examine the following nine conditions: heart failure, valvular heart disease (and specifically aortic stenosis), myocardial infarction, stroke (ischaemic and haemorrhagic) or transient ischaemic attack, peripheral vascular disease, chronic kidney disease, diabetes mellitus, as well as chronic obstructive pulmonary disease (COPD). These disease states have been further selected for investigation because interventions could be implemtented and / or tested to reduced their clinical progression. We will also quantify the occurrence of death by any cause recorded in primary care or by death certification

For peer review only

from the UK Death Register of the Office for National Statistics, which will be mapped on to 9 disease categories (Supplementary Table 2). For each condition, a list of diagnostic codes from the CALIBER code repository, including from International Classification of Diseases 10th revision (used in secondary care) and Read coding schemes (used in primary care) will be defined to comprehensively to identify diagnoses from EHRs. Incident diagnoses will be defined as the first record of that condition in primary or secondary care records from any diagnostic position. For definition of new cases, we will exclude individuals for the analysis of each condition who had a diagnosis of that condition before the patient's entry to the study. If no indication of a specific disease is recorded, then the patient will be assumed to be free from the disease. CPRD is a positive recording dataset, which reduces the likelihood of the non-recording of a clinically identified disease state.

We will create Kaplan-Meier plots for individuals identified as higher and lower predicted risk of AF to assess the event rate for non-AF outcomes censored at 10 years. We will derive the cumulative incidence rate for each outcome at 1, 5 and 10 years considering the competing risk of death, as well as death at 5 and 10 years. For each specified outcome, we will calculate the hazard ratio (HR) between higher and lower predicted risk of AF using the Fine and Gray's model with adjustment for the competing risk of death. We will also report adjusted HR where the model is adjusted for age, sex, ethnicity and the presence of any of the other outcomes at baseline. As some of the outcomes have incidence rates that are strongly associated with age (e.g. aortic stenosis) or differ by sex (e.g. heart failure),(40 41) we will conduct sub-group analyses of incidence rates for higher and lower risk individuals for each outcome by age group (30 to 64 years and ≥65 years) and sex. As some of the non-AF outcomes are more likely to occur in the setting of prevalent AF (e.g. stroke or heart failure),(10) we will also conduct a sensitivity analysis whereby people with incident AF during follow-up are excluded.

For peer review only

External validation

The CHS dataset will then be used to externally validate the model performance to assess transportability. A lack of external validation hampers the implementation of prediction models in routine clinical practice.(42) The prediction model will be saved, including the random forest structure, predictor variables, and outcome variable into a standalone file. The file will be passed onto the external international collaborators, so that they can apply the model to their local external cohort to generate predicted probability of experiencing AF at 6 months for each patient. Then the predicted probability will be compared against the observed outcome in the external cohort to assess the performance of the model. Prediction performance will be quantified by calculating the AUROC, Brier score, and by using calibration plots, and the same aforementioned clinical utility and subgroup analysis will be conducted. Performance of the prediction model will be compared with the CHA_2DS_2-VASc, C_2HEST scores.

Prediction model calculator

The full models are developed to take advantage of rich longitudinal community-based EHRs present in many high income countries. However there are other geographies (low-lower middle income countries) and care setting (emergency care, secondary care clinics) where searching for AF may be desired and an easy-to-use, simple model is preferable. From the derived prediction model, we will generate a parsimonious model based on factors with clinical rationale to predict new-onset AF over a 6 months time horizon.(10) The is will based upon the same core prinicples as detail above, but use logistic regression to ensure transparency in how prediction results are calculated. We will aim to develop a user-friendly version of a model that may be applied as a calculator in a clinical and public setting, yet have good model performance indices.

For peer review only

Software

All analysis will be conducted through R.

For peer review only

ETHICS AND DISSEMINATION

The study has been approved by CPRD (ref no: 19_076). Those handling data have completed University of Leeds information security training. All analyses will be conducted in concordance with the CPRD study dataset agreement between the Secretary of State for Health and Social Care and the University of Leeds.

The Clalit Health Services (CHS) Community Helsinki Committee and the CHS Data Utilization Committee approved the study. The study was exempt from the requirement to obtain informed consent.

The study has been registered at clinical trials.gov (NCT05837364). The study is informed by the Prognosis Research Strategy (PROGRESS) and CODE-EHR best-practice frameworks and recommendations.(42 43) The subsequent research papers will be submitted for publication in a peer-reviewed journal and will be written following TRIPOD and RECORD,(44 45) as well as the

If the model shows better prediction performance than previous models and evidence for clinical utility in analysis, it could be made readily available through EHR platforms. If the parsimonious model shows good prediction performance, the user friendly version could be accessible through the internet. Future research would be needed to assess the clinical impact of this risk model. At the point when utilisation in clinical practice is possible the applicable regulation on medicine devices will be adhered to.(46) When in clinical use, the model itself could also be reviewed and updated after incorporating evidence from the curation of more data.

For peer review only

For peer review only

Authors' contributions

RN, JW and CPG conceived the concept and planned the analysis. RN wrote the first draft, with contributions from all authors. All authors (RN, JW, CC, DH, RA, DZ, MH, TRB, LR, CPG) approved the final version and jointly take responsibility for the decision to submit the manuscript to be considered for publication.

Funding statement

RN is supported by the British Heart Foundation Clinical Research Training Fellowship (FS/20/12/34789). JW is supported by Barts Charity (MGU0504). The analysis in Clalit Health Services is funded by Israel Science Foundation Precision Membership Partnership (Grant #3543/21).

Competing Interests

None declared

For peer review only

REFERENCES

1. Wu J, Nadarajah R, Nakao YM, et al. Temporal trends and patterns in atrial fibrillation incidence: A population-based study of 3· 4 million individuals. *The Lancet Regional Health-Europe* 2022:100386.

2. Svennberg E, Engdahl J, Al-Khalili F, et al. Mass screening for untreated atrial fibrillation: the STROKESTOP study. *Circulation* 2015;131(25):2176-84.

3. Gladstone DJ, Sharma M, Spence JD. Cryptogenic stroke and atrial fibrillation. *The New England journal of medicine* 2014;371(13):1260-60.

4. Ruff CT, Giugliano RP, Braunwald E, et al. Comparison of the efficacy and safety of new oral anticoagulants with warfarin in patients with atrial fibrillation: a meta-analysis of randomised trials. *Lancet* 2014;383(9921):955-62. doi: 10.1016/S0140-6736(13)62343-0 [published Online First: 2013/12/10]

5. Hindricks G, Potpara T, Dagres N, et al. 2020 ESC Guidelines for the diagnosis and management of atrial fibrillation developed in collaboration with the European Association of Cardio-Thoracic Surgery (EACTS). *Eur Heart J* 2020 doi: 10.1093/eurheartj/ehaa612 [published Online First: 2020/08/30]

6. Ruff CT, Giugliano RP, Braunwald E, et al. Comparison of the efficacy and safety of new oral anticoagulants with warfarin in patients with atrial fibrillation: a meta-analysis of randomised trials. *The Lancet* 2014;383(9921):955-62.

7. Kirchhof P, Camm AJ, Goette A, et al. Early rhythm-control therapy in patients with atrial fibrillation. *N Engl J Med* 2020;383(14):1305-16.

8. NHS. Cardiovascular disease 2019 [updated 21 August 2019. Available from: https://www.longtermplan.nhs.uk/areas-of-work/cardiovascular-disease/.

9. Hindricks G, Potpara T, Dagres N, et al. 2020 ESC Guidelines for the diagnosis and management of atrial fibrillation developed in collaboration with the European

For peer review only

Association for Cardio-Thoracic Surgery (EACTS) The Task Force for the diagnosis and management of atrial fibrillation of the European Society of Cardiology (ESC) Developed with the special contribution of the European Heart Rhythm Association (EHRA) of the ESC. *Eur Heart J* 2021;42(5):373-498.

10. Hindricks G, Potpara T, Dagres N, et al. 2020 ESC Guidelines for the diagnosis and management of atrial fibrillation developed in collaboration with the European Association for Cardio-Thoracic Surgery (EACTS) The Task Force for the diagnosis and management of atrial fibrillation of the European Society of Cardiology (ESC) Developed with the special contribution of the European Heart Rhythm Association (EHRA) of the ESC. 2021;42(5):373-498.
11. Odutayo A, Wong CX, Hsiao AJ, et al. Atrial fibrillation and risks of cardiovascular disease, renal disease, and death: systematic review and meta-analysis. *BMJ* 2016;354
12. Gladstone DJ, Wachter R, Schmalstieg-Bahr K, et al. Screening for atrial fibrillation in the older population: a randomized clinical trial. *JAMA cardiology* 2021;6(5):558-67.
13. Halcox JP, Wareham K, Cardew A, et al. Assessment of remote heart rhythm sampling using the AliveCor heart monitor to screen for atrial fibrillation: the REHEARSE-AF study. *Circulation* 2017;136(19):1784-94.
14. Steinhubl SR, Waalen J, Edwards AM, et al. Effect of a home-based wearable continuous ECG monitoring patch on detection of undiagnosed atrial fibrillation: the mSToPS randomized clinical trial. *JAMA* 2018;320(2):146-55.
15. Kemp Gudmundsdottir K, Fredriksson T, Svennberg E, et al. Stepwise mass screening for atrial fibrillation using N-terminal B-type natriuretic peptide: the STROKESTOP II study. *EP Europace* 2020;22(1):24-32.
16. Svennberg E, Friberg L, Frykman V, et al. Clinical outcomes in systematic screening for atrial fibrillation (STROKESTOP): a multicentre, parallel group, unmasked,

randomised controlled trial. *Lancet* 2021;398(10310):1498-506. doi: 10.1016/S0140-6736(21)01637-8 [published Online First: 2021/09/02]

17. Herrett E, Gallagher AM, Bhaskaran K, et al. Data resource profile: clinical practice research datalink (CPRD). *Int J Epidemiol* 2015;44(3):827-36.

18. Himmelreich JC, Lucassen WA, Harskamp RE, et al. CHARGE-AF in a national routine primary care electronic health records database in the Netherlands: validation for 5-year risk of atrial fibrillation and implications for patient selection in atrial fibrillation screening. 2021;8(1):e001459.

19. Moons KG, Kengne AP, Woodward M, et al. Risk prediction models: I. Development, internal validation, and assessing the incremental value of a new (bio) marker. *Heart* 2012;98(9):683-90.

20. Nadarajah R, Alsaeed E, Hurdus B, et al. Prediction of incident atrial fibrillation in community-based electronic health records: a systematic review with meta-analysis. *Heart* 2021

21. Herrett E, Thomas SL, Schoonen WM, et al. Validation and validity of diagnoses in the General Practice Research Database: a systematic review. *Br J Clin Pharmacol* 2010;69(1):4-14.

22. Chisholm J. The Read clinical classification. *BMJ: British Medical Journal* 1990;300(6732):1092.

23. Hill NR, Ayoubkhani D, McEwan P, et al. Predicting atrial fibrillation in primary care using machine learning. *PLoS One* 2019;14(11):e0224582.

24. Arbel R, Hammerman A, Sergienko R, et al. BNT162b2 vaccine booster and mortality due to Covid-19. *N Engl J Med* 2021;385(26):2413-20.

For peer review only

25. Arbel R, Sergienko R, Friger M, et al. Effectiveness of a second BNT162b2 booster vaccine against hospitalization and death from COVID-19 in adults aged over 60 years. *Nat Med* 2022;28(7):1486-90.

26. Arbel R, Wolff Sagy Y, Hoshen M, et al. Nirmatrelvir use and severe Covid-19 outcomes during the Omicron surge. *N Engl J Med* 2022;387(9):790-98.

27. Hammerman A, Sergienko R, Friger M, et al. Effectiveness of the BNT162b2 vaccine after recovery from Covid-19. *N Engl J Med* 2022;386(13):1221-29.

28. Szymanski T, Ashton R, Sekelj S, et al. Budget impact analysis of a machine learning algorithm to predict high risk of atrial fibrillation among primary care patients. *EP Europace* 2022

29. Riley RD, Snell KI, Ensor J, et al. Minimum sample size for developing a multivariable prediction model: PART II-binary and time-to-event outcomes. *Stat Med* 2019;38(7):1276-96.

30. Cowan JC, Wu J, Hall M, et al. A 10 year study of hospitalized atrial fibrillation-related stroke in England and its association with uptake of oral anticoagulation. *Eur Heart J* 2018;39(32):2975-83.

31. Wu J, Alsaeed ES, Barrett J, et al. Prescription of oral anticoagulants and antiplatelets for stroke prophylaxis in atrial fibrillation: nationwide time series ecological analysis. *EP Europace* 2020;22(9):1311-19.

32. Himmelreich JC, Veelers L, Lucassen WA, et al. Prediction models for atrial fibrillation applicable in the community: a systematic review and meta-analysis. *EP Europace* 2020;22(5):684-94.

33. Routen A, Akbari A, Banerjee A, et al. Strategies to record and use ethnicity information in routine health data. *Nat Med* 2022:1-4.

For peer review only

34. Groenwold RH. Informative missingness in electronic health record systems: the curse of knowing. *Diagnostic and prognostic research* 2020;4(1):1-6.

35. Elwenspoek MM, O'Donnell R, Jackson J, et al. Development and external validation of a clinical prediction model to aid coeliac disease diagnosis in primary care: An observational study. *EClinicalMedicine* 2022;46

36. Breiman L. Random forests. *Machine learning* 2001;45(1):5-32.

37. Attia ZI, Noseworthy PA, Lopez-Jimenez F, et al. An artificial intelligence-enabled ECG algorithm for the identification of patients with atrial fibrillation during sinus rhythm: a retrospective analysis of outcome prediction. *The Lancet* 2019;394(10201):861-67.

38. Sakamoto Y, Ishiguro M, Kitagawa G. Akaike information criterion statistics. *Dordrecht, The Netherlands: D Reidel* 1986;81(10.5555):26853.

39. DeLong ER, DeLong DM, Clarke-Pearson DL. Comparing the areas under two or more correlated receiver operating characteristic curves: a nonparametric approach. *Biometrics* 1988:837-45.

40. McDonagh TA, Metra M, Adamo M, et al. 2021 ESC Guidelines for the diagnosis and treatment of acute and chronic heart failure: Developed by the Task Force for the diagnosis and treatment of acute and chronic heart failure of the European Society of Cardiology (ESC) With the special contribution of the Heart Failure Association (HFA) of the ESC. *Eur Heart J* 2021;42(36):3599-726.

41. Vahanian A, Beyersdorf F, Praz F, et al. 2021 ESC/EACTS Guidelines for the management of valvular heart disease: developed by the Task Force for the management of valvular heart disease of the European Society of Cardiology (ESC) and the European Association for Cardio-Thoracic Surgery (EACTS). *Eur Heart J* 2022;43(7):561-632.

For peer review only

42. Steyerberg EW, Moons KG, van der Windt DA, et al. Prognosis Research Strategy (PROGRESS) 3: prognostic model research. *PLoS Med* 2013;10(2):e1001381.

43. Kotecha D, Asselbergs FW, Achenbach S, et al. CODE-EHR best practice framework for the use of structured electronic healthcare records in clinical research. *BMJ* 2022;378

44. Collins GS, Reitsma JB, Altman DG, et al. Transparent Reporting of a Multivariable Prediction Model for Individual Prognosis or Diagnosis (TRIPOD) The TRIPOD Statement. *Circulation* 2015;131(2):211-19.

45. Nicholls SG, Quach P, von Elm E, et al. The reporting of studies conducted using observational routinely-collected health data (RECORD) statement: methods for arriving at consensus and developing reporting guidelines. *PLoS One* 2015;10(5):e0125620.

46. Kramer DB, Xu S, Kesselheim AS. Regulation of medical devices in the United States and European Union. The Ethical Challenges of Emerging Medical Technologies: Taylor and Francis 2020:41-49.

For peer review only

Patient consent for publication

Not required

Ethics Approval

Permissions for the CPRD-GOLD and CPRD-AURUM datasets were obtained from CPRD (ref no: 19_076). The study was approved by CPRD ethical approval committee. The Clalit Health Services (CHS) Community Helsinki Committee and the CHS Data Utilization Committee approved the study. The study was exempt from the requirement to obtain informed consent.

Word Count

3999

Figure Legend

Figure 1, A schematic representation comparing current AF screening approaches, which focus on stroke prevention, with a broader approach to AF screening that considers that individuals eligible for AF screening will be at risk of multiple outcomes beyond stroke.

Figure 2. A schematic representation of a multivariable logistic regression model or random forest model using data from electronic health records to provide risk prediction for incident AF.

For peer review only

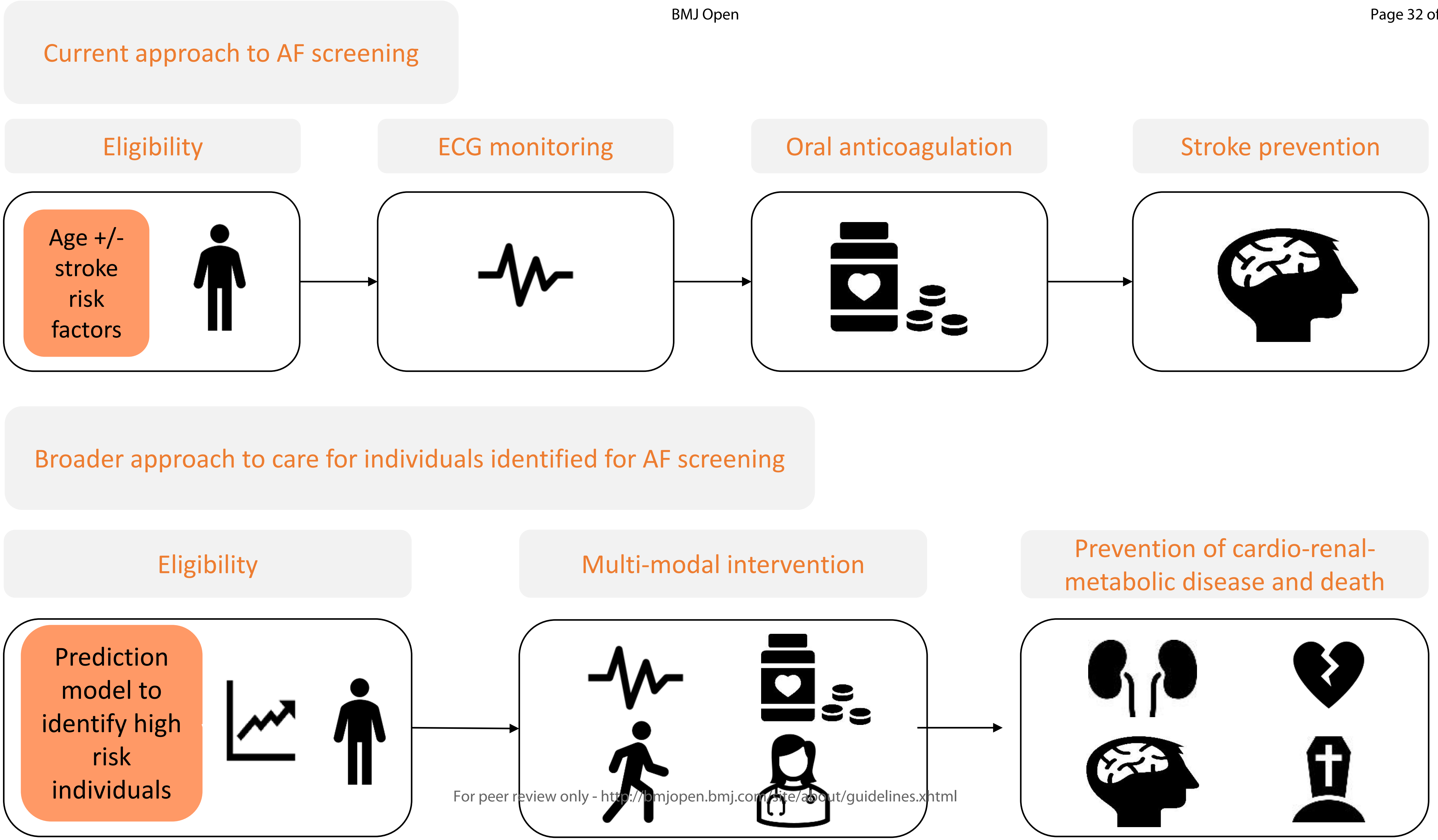
Current approach to AF screening
Eligibility
ECG monitoring
Oral anticoagulation
Stroke prevention
Age +/- stroke risk factors
Broader approach to care for individuals identified for AF screening
Eligibility
Multi-modal intervention
Prevention of cardio-renal-metabolic disease and death
Prediction model to identify high risk individuals

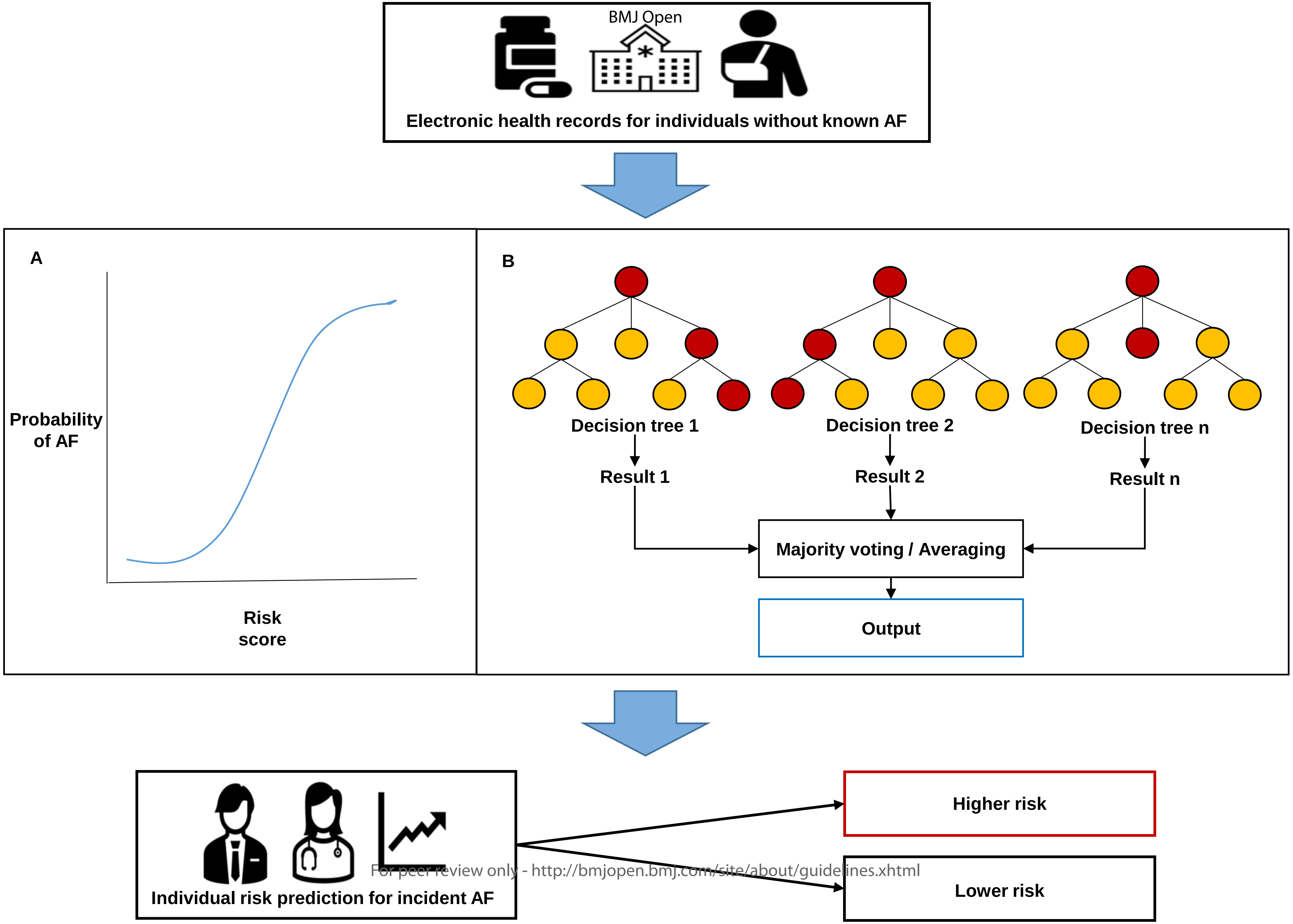

For peer review only - http://bmjopen.bmj.com/site/about/guidelines.xhtml

Supplementary Appendix

Risk of atrial fibrillation and association with other diseases: protocol of the derivation and international external validation of a prediction model using nationwide population-based electronic health records

Ramesh Nadarajah, Jianhua Wu, Ronen Arbel, Moti Haim, Doron Zahger, Talish Razi Benita, Lior Rokach, Campbell Cowan, Chris P Gale

Supplementary Table 1. Baseline demographic and comorbidity variables used in algorithms tested for predicting incident AF in community-based electronic health records 2

Supplementary Table 2. Definition of disease categories for causes of deaths 4

Supplementary Figure 1. Design process leading to selection of non-AF outcomes to assess for association with predicted AF risk 5

For peer review only

Supplementary Table 1. Baseline demographic and comorbidity variables used in algorithms tested for predicting incident AF in community-based electronic health records

Algorithm	Demographics	Comorbidities
$CHADS_2$	Age	Hypertension, CHF, diabetes mellitus, CVA
CHA_2DS_2-VASc	Age, sex	Hypertension, CHF, stroke/TIA/thromboembolism, vascular disease
CHARGE-AF	Age, race, smoking status	Anti-hypertensive medication, MI, CHF, DM
C_2HEST	Age	Hypertension, ischaemic heart disease, CHF, COPD, thyroid disease
HATCH	Age	Hypertension, CHF, stroke/TIA, COPD
InGef	Age, sex	Anti-hypertension medication, heart failure medication, chronic kidney disease, disorderd of lipoprotein metabolism and other lipidaemias, pulmonary heart diseases cardiac arrhythmias, other cerebrovascular disease, diverticular disease of intestine, dorsalgia, breathing abnormalities
MHS	Age, sex	Anti-hypertensive medication, MI, CHF, peripheral vascular disease, inflammatory disease in a female, COPD
NHIRD	Age (years), age group, sex	Hypertension, CHF, COPD, rheumatological disease, dyslipidaemia, DM, CVA or TIA, sleep disorder, cancer, hyperthyroidism, vascular disease, gout, CKD or ESRD, anaemia
NHIS-NSC*	Age, sex, smoking (pack-year), alcohol	Hypertension, CHF, MI, vascular disease, stroke/TIA, COPD
Pfizer-AI	Age, sex, race, smoking status	Hypertension, anti-hypertensive medication, CHF, congenital heart disease, MI, LVH, type 1 DM, type 2 DM
Taiwan AF	Age, sex, alcohol excess	Hypertension, CHF, IHD, ESRD

AF, Atrial Fibrillation; $CHADS_2$, Congestive heart failure, Hypertension, Age >75, Diabetes mellitus, prior Stroke or transient ischemic attack [2 points]; CHA_2DS_2-VASc, Congestive heart failure, Hypertension, Age >75 [2 points], Stroke/transient ischemic attack/thromboembolism [2 points]; CHARGE-AF, Cohorts for Heart and Aging Research in Genomic Epidemiology; C_2HEST, Coronary artery disease / Chronic obstructive pulmonary disease [1 point each], Hypertension, Elderly (Age ≥75, 2 points), Systolic heart failure, Thyroid disease (hyperthyroidism); CHF, chronic heart failure; CKD, chronic kidney disease; COPD, chronic obstructive pulmonary disease; CPRD, Clinical Practice Research Datalink; CVA, cerebrovascular accident; DM, diabetes mellitus; ESRD, end-stage renal disease; HATCH, Hypertension, Age, stroke or Transient ischemic attack, Chronic obstructive pulmonary disease, Heart failure; IHD, ischaemic heart disease; LVH, left ventricular hypertrophy; MHS, Maccabi Healthcare Services; MI, myocardial infarction; NHIRD, National Health Insurance Research Database; NHIS-HEALS, National Health Insurance Service - Health screening Cohort; NHIS-NSC, National Health Insurance Service-based National Sample Cohort; TIA, transient ischaemic attack.

For peer review only - http://bmjopen.bmj.com/site/about/guidelines.xhtml

* In Kim 2020 prediction model development using machine learning was completed both with and without the predictor $PM_{2.5}$ - which is fine particular matter air pollution. In this analysis we have only included the model without $PM_{2.5}$ as it is judged not to be a predictor that would be routinely available in primary care or population EHR.

For peer review only

For peer review only - http://bmjopen.bmj.com/site/about/guidelines.xhtml

Supplementary Table 2. Definition of disease categories for causes of deaths

Causes of death	Code
**Cardiovascular disorders**	ICD chapter 'Diseases of the circulatory system' (code range: I00–I99), excluding codes relating to infections or cerebrovascular disease.
**Cerebrovascular disorders**	ICD chapter 'Diseases of the circulatory system' (I60-I69)
**Neoplasms**	ICD chapter 'Neoplasms' (C00–D48).
**Infections**	Infectious and parasitic diseases, respiratory infections, urinary tract infections, and cellulitis, as defined by individual codes as Conrad et al.
**Chronic respiratory diseases**	Individual codes Conrad et al.
**Digestive diseases**	ICD chapter 'Diseases of the digestive system' (K00–K93), excepting selected codes categorized as infections.
**Mental and neurological disorders**	ICD chapter 'Mental and behavioral disorders' (F00–F99) and ICD chapter 'Diseases of the nervous system' (G00–G99)
**Injuries**	ICD chapters 'Injury, poisoning and certain other consequences of external causes' (S00–T98) and 'External causes of morbidity and mortality' (V01–Y98)
**Kidney diseases**	ICD sub-chapters 'Renal failure' (N17-N19), 'Glomerular diseases' (N00-N08), 'Renal tubulo-interstitial diseases' (N10-N16), 'Other disorders of kidney and ureter' (N25-N29)

To categorise cause of death as infections or chronic respiratory diseases we used the same codelists as Conrad N, Judge A, Canoy D, et al. Temporal trends and patterns in mortality after incident heart failure: a longitudinal analysis of 86 000 individuals. *JAMA cardiology* 2019;4(11):1102-11

For peer review only

For peer review only - http://bmjopen.bmj.com/site/about/guidelines.xhtml

Supplementary Figure 1. Design process leading to selection of non-AF outcomes to assess for association with predicted AF risk

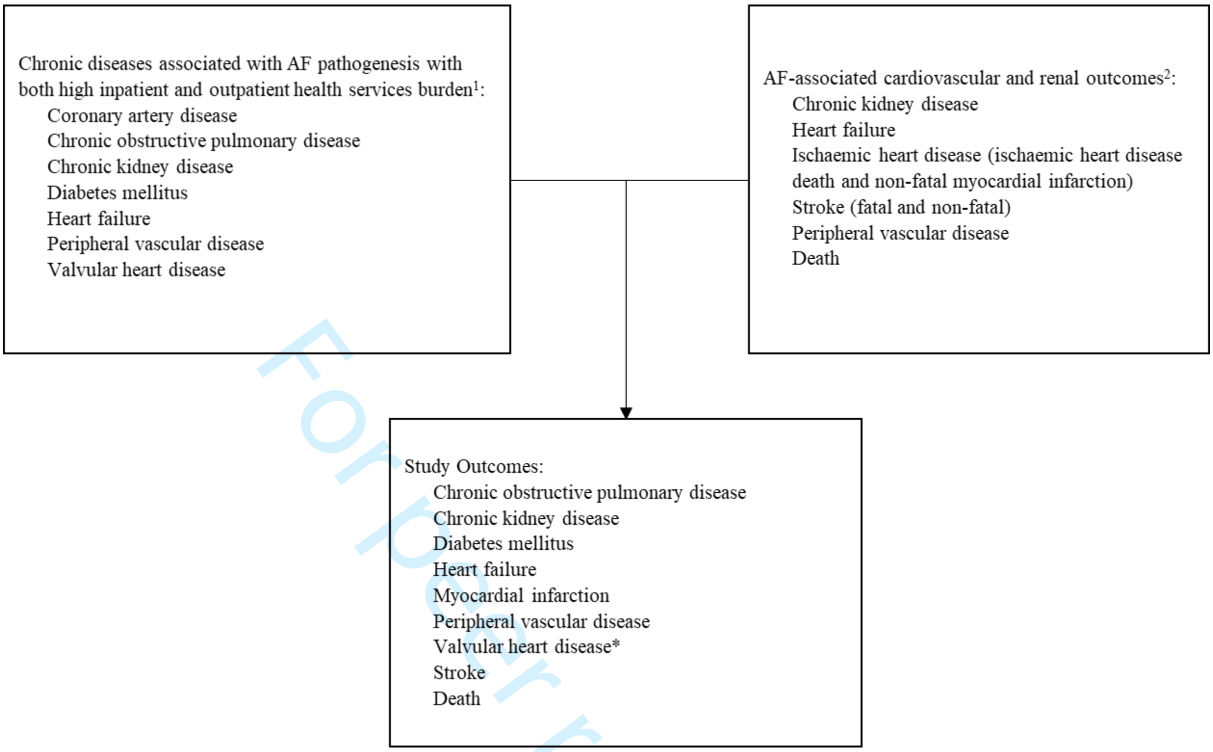

[1] Hindricks G, Potpara T, Dagres N, et al. 2020 ESC Guidelines for the diagnosis and management of atrial fibrillation developed in collaboration with the European Association for Cardio-Thoracic Surgery (EACTS) The Task Force for the diagnosis and management of atrial fibrillation of the European Society of Cardiology (ESC) Developed with the special contribution of the European Heart Rhythm Association (EHRA) of the ESC. 2021;42(5):373-498.

[2] Odutayo A, Wong CX, Hsiao AJ, et al. Atrial fibrillation and risks of cardiovascular disease, renal disease, and death: systematic review and meta-analysis. *BMJ* 2016;354

* Aortic stenosis was further specified in addition to valvular heart disease given the increasing availability and randomised controlled trial evidence for earlier treatment, and increasing therapeutic options across operative risk profiles (Vahanian A, Beyersdorf F, Praz F, et al. 2021 ESC/EACTS Guidelines for the management of valvular heart disease: developed by the Task Force for the management of valvular heart disease of the European Society of Cardiology (ESC) and the European Association for Cardio-Thoracic Surgery (EACTS). *Eur Heart J* 2022;43(7):561-632.)

For peer review only - http://bmjopen.bmj.com/site/about/guidelines.xhtml
